# GAP43-dependent mitochondria transfer from astrocytes enhances glioblastoma tumorigenicity

**DOI:** 10.1038/s43018-023-00556-5

**Published:** 2023-05-11

**Authors:** Dionysios C. Watson, Defne Bayik, Simon Storevik, Shannon Sherwin Moreino, Samuel A. Sprowls, Jianhua Han, Mina Thue Augustsson, Adam Lauko, Palavalasa Sravya, Gro Vatne Røsland, Katie Troike, Karl Johan Tronstad, Sabrina Wang, Katharina Sarnow, Kristen Kay, Taral R. Lunavat, Daniel J. Silver, Sahil Dayal, Justin Vareecal Joseph, Erin Mulkearns-Hubert, Lars Andreas Rømo Ystaas, Gauravi Deshpande, Joris Guyon, Yadi Zhou, Capucine R. Magaut, Juliana Seder, Laura Neises, Sarah E. Williford, Johannes Meiser, Andrew J. Scott, Peter Sajjakulnukit, Jason A. Mears, Rolf Bjerkvig, Abhishek Chakraborty, Thomas Daubon, Feixiong Cheng, Costas A. Lyssiotis, Daniel R. Wahl, Anita B. Hjelmeland, Jubayer A. Hossain, Hrvoje Miletic, Justin D. Lathia

**Affiliations:** 1 Lerner Research Institute, Cleveland Clinic, Cleveland, OH USA; 2 Case Comprehensive Cancer Center, Cleveland, OH USA; 3 University Hospitals Cleveland Medical Center, Cleveland, OH USA; 4 School of Medicine, Case Western Reserve University, Cleveland, OH USA; 5 Department of Biomedicine, University of Bergen, Bergen, Norway; 6 Department of Neurology, Haukeland University Hospital, Bergen, Norway; 7 Cleveland Clinic Lerner College of Medicine, Case Western Reserve University, Cleveland, OH USA; 8 Medical Scientist Training Program, Case Western Reserve University, Cleveland, OH USA; 9 Department of Radiation Oncology, University of Michigan, Ann Arbor, MI USA; 10 Department of Neurology, Molecular Neurogenetics Unit-West, Massachusetts General Hospital, Boston, MA USA; 11 Stipe Therapeutics, Aarhus, Denmark; 12 University of Bordeaux, INSERM, BRIC, Pessac, France; 13 Cancer Metabolism Group, Department of Cancer Research, Luxembourg Institute of Health, Luxembourg, Luxembourg; 14 University of Alabama at Birmingham, Birmingham, AL USA; 15 Rogel Cancer Center, University of Michigan, Ann Arbor, MI USA; 16 Cancer Biology Graduate Program, University of Michigan, Ann Arbor, MI USA; 17 NorLux Neuro-Oncology Laboratory, Department of Cancer Research, Luxembourg Institute of Health, Luxembourg, Luxembourg; 18 University of Bordeaux, CNRS, IBGC, Bordeaux, France; 19 Department of Molecular & Integrative Physiology, University of Michigan, Ann Arbor, MI USA; 20 Department of Internal Medicine, Division of Gastroenterology and Hepatology, University of Michigan, Ann Arbor, MI USA; 21 Department of Pathology, Haukeland University Hospital, Bergen, Norway; 22 Present Address: Sylvester Comprehensive Cancer Center, Miller School of Medicine, University of Miami, Miami, FL USA

**Keywords:** Cancer microenvironment, CNS cancer, Cancer, Cell division, Mitochondria

## Abstract

The transfer of intact mitochondria between heterogeneous cell types has been confirmed in various settings, including cancer. However, the functional implications of mitochondria transfer on tumor biology are poorly understood. Here we show that mitochondria transfer is a prevalent phenomenon in glioblastoma (GBM), the most frequent and malignant primary brain tumor. We identified horizontal mitochondria transfer from astrocytes as a mechanism that enhances tumorigenesis in GBM. This transfer is dependent on network-forming intercellular connections between GBM cells and astrocytes, which are facilitated by growth-associated protein 43 (GAP43), a protein involved in neuron axon regeneration and astrocyte reactivity. The acquisition of astrocyte mitochondria drives an increase in mitochondrial respiration and upregulation of metabolic pathways linked to proliferation and tumorigenicity. Functionally, uptake of astrocyte mitochondria promotes cell cycle progression to proliferative G2/M phases and enhances self-renewal and tumorigenicity of GBM. Collectively, our findings reveal a host–tumor interaction that drives proliferation and self-renewal of cancer cells, providing opportunities for therapeutic development.

## Abstract (web summary)

Watson et al. demonstrate that astrocyte mitochondria can be horizontally transferred to glioblastoma cells in a GAP43-dependent manner, leading to changes in mitochondrial respiration and metabolism that promote proliferation and tumor growth.

## Main

Mitochondria are vital in cell metabolism through their role in generating ATP via oxidative phosphorylation; yet, they have also been implicated in numerous other cellular functions, including apoptotic cell death, inflammation, stem cell differentiation and autophagy^1,2^. After the discovery of the Warburg effect identifying conversion of glucose to lactate in the presence of oxygen to fuel cancer growth^3^, aerobic glycolysis has been assumed to be the main energy pathway for cancer cells^4^, including in glioblastoma (GBM)^5^, the most common primary adult brain tumor. However, recent reports suggest a more complex situation where mitochondrial respiration is also an alternative energy source^6,7^. The existence of multiple metabolic phenotypes is in line with the cellular heterogeneity of this disease^8–10^.

While this heterogeneity may arise from variation in cell-intrinsic metabolic regulation, it may also arise via interaction with the tumor microenvironment (TME), namely by transfer of metabolic signals, including mitochondria themselves, from the TME to tumor cells. In many contexts, dynamic microenvironmental interactions have been shown to be major drivers of tumor growth and therapeutic resistance in GBM^11–13^, while the underlying direct cell–cell communication mechanisms remain poorly understood.

Transfer of mitochondria has been demonstrated between different cell types and via different routes. Mitochondria transfer by extracellular vesicles from astrocytes to neurons after stroke has been documented in vitro and in vivo^14^, identifying a CD38/cADP-dependent mechanism using calcium signaling. Extracellular vesicles as a mode of mitochondria transfer has also been shown for mesenchymal stem cells^15^, indicating that transfer by secretion is a viable route for this organelle. In other studies, mitochondria transfer mechanisms independent of secreted particles have been proposed^16–18^. Intercellular membrane protrusions known as tunneling nanotubes have been implicated in organelle transport^19^ and more recently in mitochondria transfer^16–18^. By connecting cells over distances that may exceed 100 μm (refs. ^20,21^), tunneling nanotubes are prime avenues for intercellular cross-talk^22^; composed of components like actin and microtubules^22^, they maintain a close cytoskeletal relationship^22^.

Tumor microtubes (MTs) are similar structures; however, they are thicker and more stable than tunneling nanotubes and can reach lengths of more than 500 μm in vivo^23^. MTs have so far only been described in glioma, particularly in GBM, and have been identified as an important mode of intercellular communication, invasion and therapy resistance^24–27^. Growth-associated protein 43 (GAP43), a neuronal protein known for its important role in axon guidance^28^, is a major structural protein of MTs. Knockdown of GAP43 in GBM cells reduced MT formation and tumorigenicity in vivo^24^. This work highlighted the relevance of the interconnected network of GBM cells formed by GAP43^+^ MTs for tumor growth. In a recent study, mitochondria were reported to be exchanged in vitro among GBM cells interconnected via MTs^29^.

Given the reports of horizontal mitochondria transfer in the brain across distinct cell types under pathologic conditions^14^, we hypothesized that GBM also acquires functional mitochondria from non-malignant cells in the TME, comprising an unexplored layer of metabolic heterogeneity. Here, we show that GBM cells acquire host mitochondria from astrocytes through a contact-dependent mechanism facilitated by GAP43^+^ structures consistent with MTs, resulting in enhanced metabolic activity and augmented tumorigenicity. Our findings identify mitochondria transfer from the TME as a fundamental protumorigenic mechanism in GBM.

## Results

### GBM acquires mitochondria from the microenvironment

**Fig. 1:**
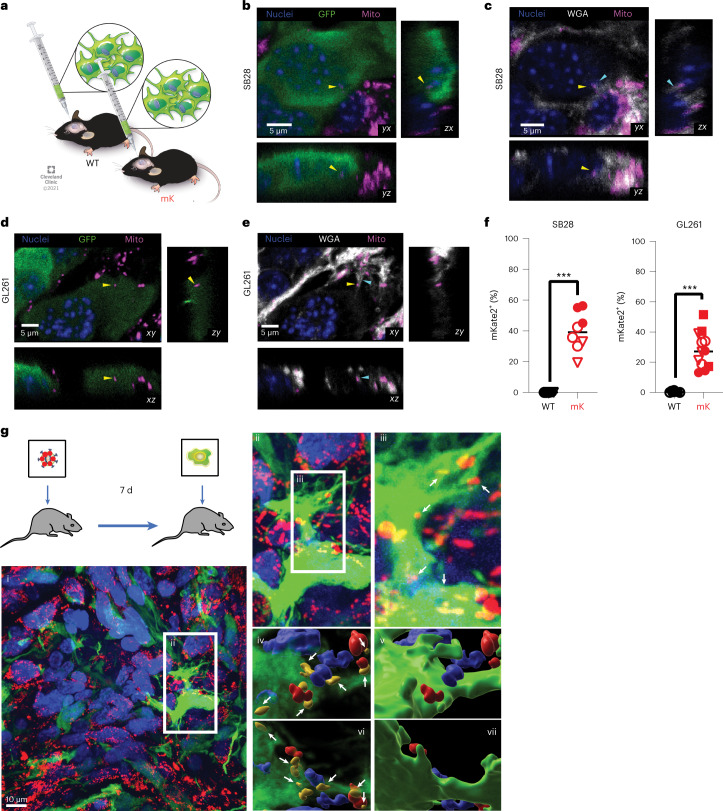
GBM cells acquire host mitochondria from the TME. **a**, GFP-expressing GL261 and SB28 cells were implanted intracranially into wild-type (WT) and mito::mKate2 (mK) mice, and tumors were analyzed at humane endpoint. **b**–**e**, Single focal planes (*xy*) and *z*-stack orthogonal reconstructions (*xz*, *yz*) at areas of SB28 (**b**,**c**) and GL261 (**d**,**e**) tumor–host cell interfaces. Yellow arrowheads indicate host mKate2^+^ mitochondria (Mito) within recipient tumor cells. Cyan arrowheads indicate WGA-labeled tether-like structures connecting tumor and host cells. **f**, Three-dimensional confocal imaging segmentation-based estimation of mKate2^+^GFP^+^ GBM cell frequency from two to three visual fields from *n* = 3 (SB28) and *n* = 2 (GL261) WT and *n* = 3 (SB28) and *n* = 4 (GL261) mK mice; ***P = 0.0003 (SB28) and *P* < 0.0001 (GL261). Data were analyzed by two-tailed *t*-test. See also Extended Data Fig. 1 for additional data, including technical controls. **g**, Mitochondria transfer between the TME (mitoDsRed^+^) and human GBM cells (GFP^+^) in vivo. MitoDsRed lentivirus was injected into the brains of nude rats. Seven days later, human P3 GFP^+^ GSCs were injected into the same area. Confocal microscopy images of P3 GFP^+^ xenograft tumors are representative of at least six ×100 images across three biologically independent animals. Details of the area along the tumor surface are shown in (i), with colocalization of GFP^+^ and mitoDsRed^+^ signal in (ii) and (iii). A 3D reconstruction of the mitoDsRed^+^ mitochondria (white arrows) seen from above, without (iv) and with (v) the GFP^+^ cell borders, is shown. From below, the mitoDsRed^+^ mitochondria are also visible in (vi) and reside within the cell in (vii). ii, ×1.5 magnification; iii–vii, ×3 magnification. Source data

**Extended Data Fig. 1:**
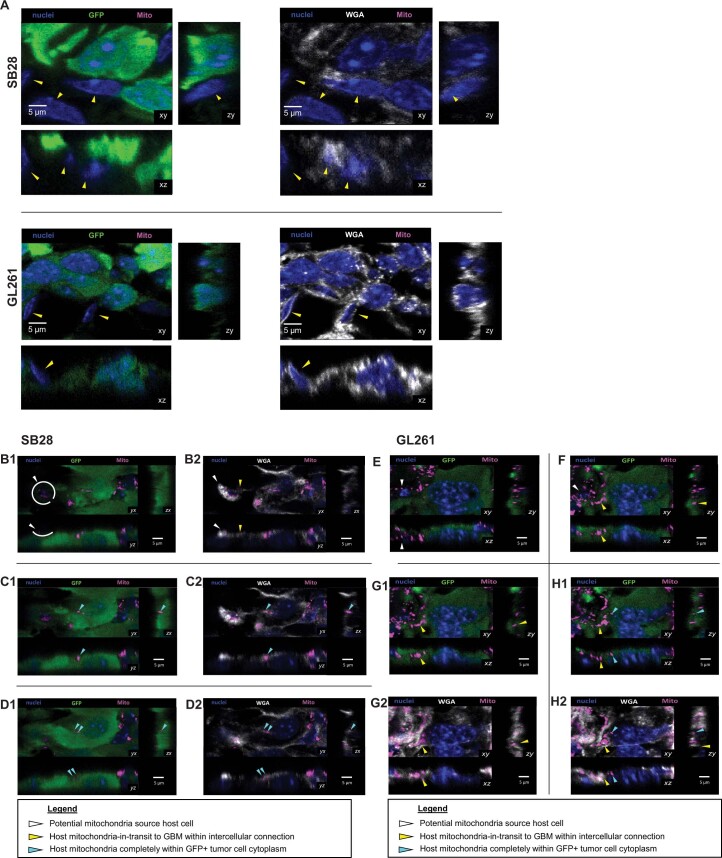
Additional data for *in vivo* transfer of host mitochondria to orthotopic mouse GBM tumors. (**A**) GFP-expressing GL261 and SB28 mouse GBM cells were implanted intracranially into wildtype mice (controls for Fig. 1B-F), and tumors were analyzed at animal humane endpoint. Representative confocal microscopy data from orthotopic SB28 and GL261 tumors in wildtype mice are shown. Yellow arrowheads point to GFP^negative^ (non-tumor) host cells. Shown are single focal planes (xy), as well as z-stacks (zy and xz). (**B**→**D**) and (**E**→**H**) Mitochondria in-transit from host to GBM cells in orthotopic tumors. Sequential confocal planes (xy) and accompanying orthogonal reconstructions (zy, xz) of SB28 (**B**→**D**) and GL261 (**E**→**H**) GBM tumors in mice, demonstrating an intercellular connection between a mito::mKate2^+^ host cell (white arrowheads) and GFP^+^ tumor cell. Host mKate2^+^ mitochondria within the intercellular connection are indicated by yellow arrowheads. The mitochondria at the end of the connection are surrounded by GFP signal, corresponding to incorporation in the recipient tumor cell cytoplasm (cyan arrowhead).

**Extended Data Fig. 2:**
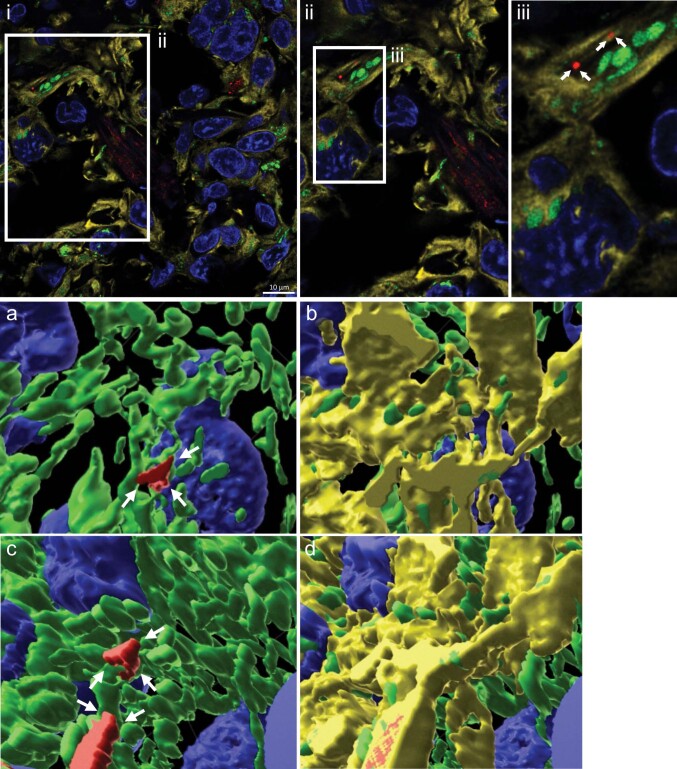
Mitochondria transfer from the TME to human GBM models is observed *in vivo*. Mitochondria transfer between the TME (mitoDsRed + ) and tumor cells (mitoGFP+ and nestin + ) *in vivo*. Confocal microscopy of GG16 tumors immunostained with human-specific nestin antibodies, representative of at least four 100X images across 3 biologically independent animals. (i). Details of area along the tumor surface with co-localization of nestin + (yellow), mitoGFP+ and mitoDsRed+ signal (ii and iii). 3D reconstruction of the mitoDsRed+ mitochondria seen from above, without (a) and with (b) the nestin+ cell borders. From below, the mitoDsRed+ mitochondria are also visible (c) and reside within the cell (d). ii, 1.5x magnification; iii and a-d, 3x magnification.

To assess whether non-malignant host cells could transfer mitochondria to GBM in vivo, we orthotopically implanted green fluorescent protein (GFP)-expressing syngeneic mouse GBM models (SB28 and GL261) into transgenic C57BL/6 mice expressing a mitochondria-localized mKate2 fluorophore fused to the localization peptide of cytochrome *c* oxidase 8 (mito::mKate2 mice^30^; Fig. 1a). Confocal microscopy of GBM tumors from mito::mKate2 mice revealed mKate2^+^ puncta within 15–60% of GFP^+^ GBM cells (Fig. 1b–f and Extended Data Fig. 1), demonstrating that host cell mitochondria were acquired in vivo by orthotopic GBM tumor cells. By co-staining tissue sections with wheat germ agglutinin (WGA) to highlight glycoprotein-rich structures^31^, we observed host mKate2^+^ mitochondria in transit at host–tumor interfaces along intercellular connections between GFP^+^ GBM cells and GFP^–^ host cells (Extended Data Fig. 1). To determine whether mitochondria transfer occurred in the context of human GBM in vivo, we first injected a high-titer mitoDsRed lentivirus into the brain of nude rats to transduce normal brain cells with a mitochondria fluorescent tag. After 7 d, P3 GFP^+^ human-derived GBM stem-like cells (GSCs) were injected in the same location (Fig. 1g). High-resolution confocal images verified mitochondria transfer from stromal cells to tumor cells, supporting our mouse in vivo data (Fig. 1g). When reconstructed in three dimensions, the mitoDsRed signal was present within acceptor cells positive for GFP. We verified these results in a second human-derived GSC line GG16 transduced with mito-GFP and by immunostaining with a human-specific nestin antibody (Extended Data Fig. 2).

**Extended Data Fig. 3:**
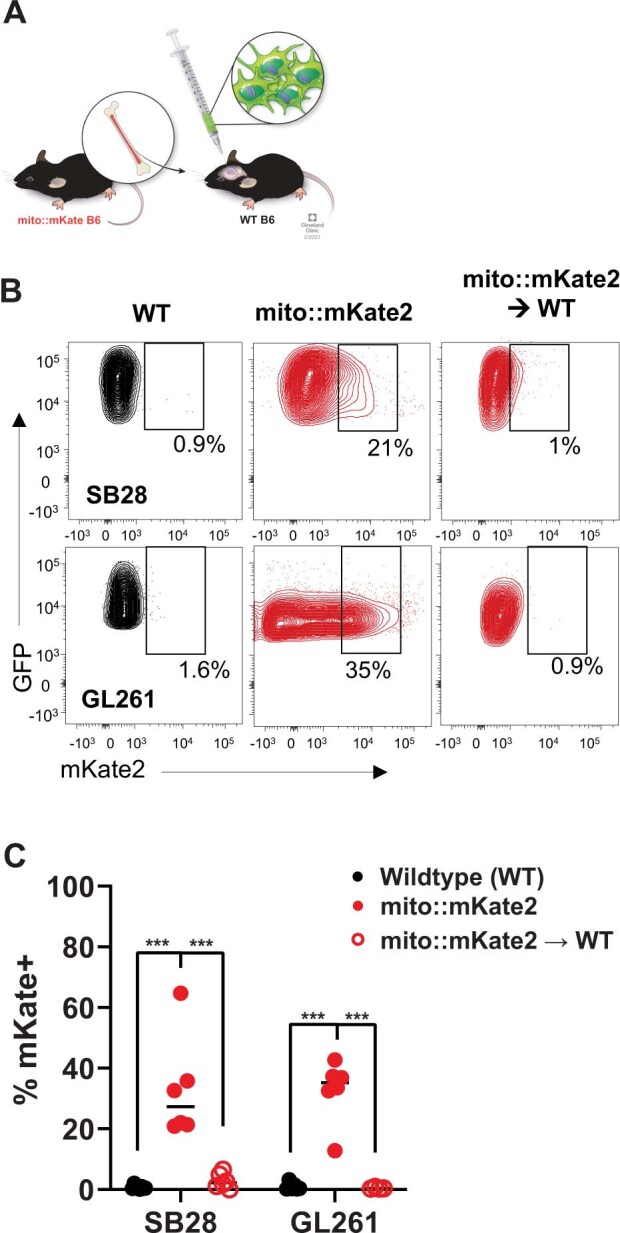
Brain-resident cells transfer mitochondria to GBM cells *in vivo*. (**A-C**) GFP-expressing SB28 and GL261 cells were implanted intracranially into wild-type (WT), mKate2::mito (mKate2) or WT mice with mito::mKate2 bone marrow (mito::mKate2→WT). (**B**) Representative contour plots and (**C**) aggregate data of relative frequency of mito::mKate2^+^GFP^+^ GBM cells by flow cytometry; n = SB28 (8 WT, 6 mito::mKate2, and 6 mito::mKate2→WT) and GL261 (6 WT, 6 mito::mKate2, and 4 mito::mKate2→>WT) mice per group. *** p < 0.0001, 2-way ANOVA with Tukey correction for multiple comparisons. Mitochondria transfer rate is negligible in mito::mKate2→WT mice, in which brain-resident cells do not express mito::mKate2. Source data

Having observed mitochondria transfer from the TME to mouse and human GBM models, we proceeded to interrogate the identity of the host mitochondria donor cells. GBM tumors in mouse models and humans are known to have substantial infiltration by both brain-resident glia and peripheral immune cells that transmigrate into the TME^13^. We established orthotopic GBM tumors in wild-type C57BL/6 mice that had first received lethal irradiation with subsequent bone marrow reconstitution from mito::mKate2 mice (mito::mKate2→WT) to restrict mito::mKate2 expression to bone marrow-derived immune cells (Extended Data Fig. 3a). Analysis of single-cell suspensions by flow cytometry indicated negligible host mitochondria transfer to GFP^+^ GBM cells in mito::mKate2→WT mice, while 20–60% of GFP^+^ GBM cells were mKate2^+^ in mito::mKate2 mice (Extended Data Fig. 3b,c). Taken together, these data suggest that brain-resident cells, not tumor-infiltrating immune cells, were the major donors of mitochondria to GBM cells in vivo.

**Fig. 2:**
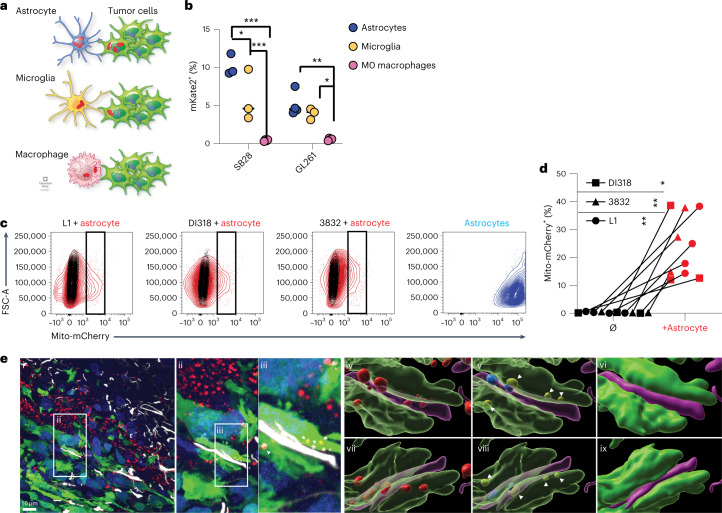
GBM cells acquire mitochondria from astrocytes. **a**, GBM cells were cocultured with astrocytes, microglia or macrophages from mK mice for 2 h, and mitochondria transfer was analyzed by flow cytometry. **b**, Relative frequency of mKate2^+^GFP^+^ GBM cells in co-cultures; *n* = 3 (SB28) and 4 (GL261) independent experiments; *P < 0.05, **P < 0.01, ***P < 0.001. Data were analyzed by two-way analysis of variance (ANOVA). **c**,**d**, Human-derived GSCs were cocultured with immortalized mito-mCherry^+^ human astrocytes for 4 d. Mito-mCherry^+^ GSCs (black rectangle gate) were quantified by flow cytometry (**c**), as summarized in **d**; *n* = 3 (3832), 3 (DI318) and 4 (L1) independent experiments. Data were analyzed by two-way ANOVA; *P* = 0.006 (L1), 0.03 (DI318) and 0.007 (3832). **e**, Mitochondria transfer between astrocytes (mitoDsRed^+^ and GFAP^+^) and human GBM cells (GFP^+^) in vivo. Confocal microscopy of a GFP^+^ P3 xenograft tumor immunostained with antibodies to GFAP (white color) was used to visualize astrocytes. Images are representative of at least six ×100 images across three biologically independent animals. Mitochondria transfer is highlighted in an invasive tumor area with colocalization of GFP^+^ and mitoDsRed^+^ signal in (i). Images in (ii) and (iii)–(ix) represent ×1.5 and ×3 magnifications, respectively. A 3D reconstruction of the mitoDsRed^+^ mitochondrial signal both within and around the GFP^+^ and GFAP^+^ surfaces is shown in (iv). The mitoDsRed^+^ mitochondria colocalized within the GFP^+^ tumor cell borders (yellow) and within the purple reconstructed GFAP^+^ astrocytic processes (blue), seen from above without (v) and with (vi) GFP^+^ and GFAP^+^ cell borders. From below, mitoDsRed^+^ mitochondria are also visible in (vii) and reside within the GFP^+^ and GFAP^+^ regions in (viii) and (ix). Source data

**Extended Data Fig. 4:**
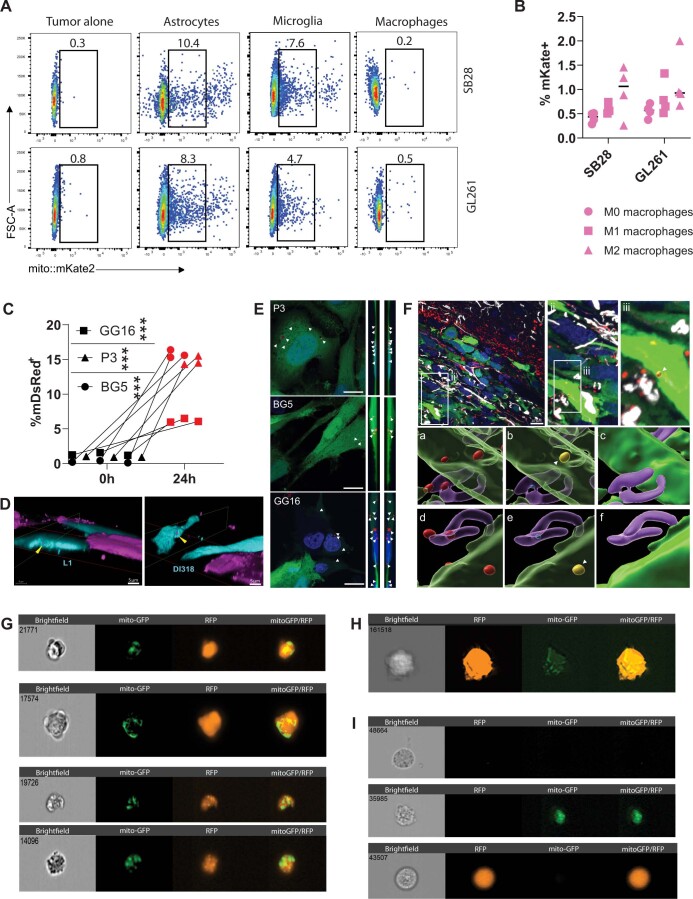
Additional data for in vitro mitochondria transfer in mouse and diverse patient-derived GBM models. (**A**) Representative flow cytometry dot plots depicting mitochondria transfer frequency to mouse GBM cells from mito::mKate2 donor cells, summarized in Fig. 2B. (**B**) Mitochondria transfer from polarized (M1, M2) or non-polirized (M0) bone marrow-derived macrophages, assessed by flow cytometry. n = 4 independent experiments. Two-way ANOVA. (**C**) Flow cytometry of mitochondria transfer between mDsRed+ astrocytes and P3/BG5/GG16 GFP + cells at 24 h; mean ± SD. n = 3 independent experiments. *** p < 0.0001 (2-tailed t-test). (**D**) 3D reconstructions of confocal microscopy from patient-derived GBM and astrocyte co-cultures. mito-mCherry astrocytes (magenta); cyan cells CellTrace-labelled GSCs (cyan). Perpendicular cutaway planes (red and green outlines) reveal internalized astrocyte-derived mito-mCherry^+^ mitochondria in GSCs (yellow arrowheads). (**E**) Confocal microscopy of mitochondria transfer in P3/BG5/GG16 GFP + cells with mDsRed+ astrocyte mitochondria (arrows); representative of at least four 100x images across 3 biologically independent animals for each cell line. Scale bars 10 mm. Z stack locates mDsRed+ mitochondria (arrows) within acceptor cell cytoplasm, from the left side (i) and the right (ii). **(F)** Mitochondria transfer between astrocytes (mitoDsRed^+^GFAP^+^) and human GBM cells (GFP^+^) *in vivo*. Confocal microscopy of GFP^+^ P3 xenograft tumor immunostained for GFAP (white) to visualize astrocytes; representative of at least six 100X images across 3 biologically independent animals. (i) Mitochondria transfer highlighted at invasive tumor area with colocalization of GFP + and mitoDsRed+ signal (ii and iii, higher magnification). 3D reconstruction of the mitoDsRed+ mitochondrial signal within and around GFP + and GFAP + surfaces (a). mitoDsRed+ mitochondria colocalize within GFP + tumor cells (yellow) and within the purple reconstructed GFAP + astrocytic processes (blue), seen from above without (b) and with (c) GFP + and GFAP + cell borders. From below, mitoDsRed+ mitochondria are also visible (d) and reside within the GFP + and GFAP + regions (e-f). Scale bars 10 µm. (**G-H**) ImageStream depicting transferred mito-GFP astrocyte mitochondria to co-cultured (**G**) D456 and (**H**) JX22 patient-derived RFP^+^ GBM cells, representative across >imaged 100 cells per cell type and model. (**I)** Single-cell culture controls demonstrating the specificity of the RFP (tumor) and GFP (astrocyte mitochondria) signals. *Top to bottom:* Non-transduced GBM cells; mito-GFP astrocytes; RFP GBM cells. Source data

To further elucidate the identity of the predominant mitochondria donor cell populations, we cocultured prevalent tumor-infiltrating cell types with GFP^+^ GBM cells at a 1:2 donor:recipient ratio. After 2 h, we assessed the percentage of mKate2^+^ cells as a marker of mitochondria transfer (schematized in Fig. 2a). Consistent with our in vivo observations, we found that brain-resident glia (astrocytes and microglia) donated significantly more mitochondria on a per-cell basis than bone marrow-derived macrophages, with astrocytes having higher transfer rates (Fig. 2b and Extended Data Fig. 4a). While further polarization of macrophages to an M2- or M1-like phenotype potentially favored increased mitochondria transfer, the degree of transfer was, on average, five- to tenfold less than that observed with the brain-resident glia in vitro (Extended Data Fig. 4b).

**Extended Data Fig. 5:**
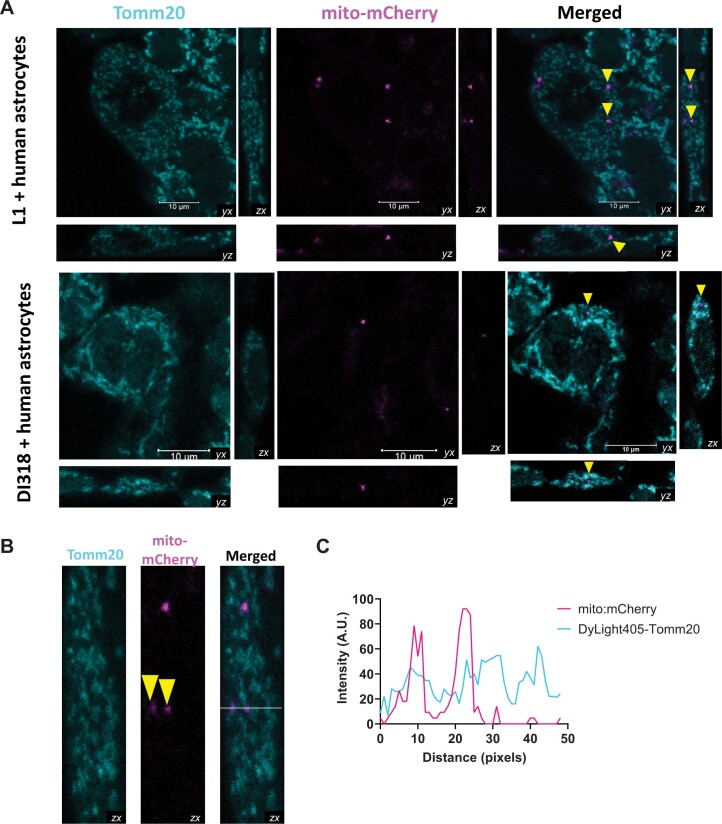
Astrocyte mito-mCherry^+^ structures internalized by patient-derived GSCs comprise Tomm20^+^ mitochondria. Co-cultures of patient-derived GSCs (L1 and DI318) and mito-mCherry (magenta) human astrocytes were fixed and stained for the mitochondrial outer membrane protein Tomm20 (cyan). (**A**) Confocal microscopy, including z-stacks, depicting distribution of internalized astrocyte-derived mito-mCherry^+^ mitochondria (yellow arrowheads) in relation with intrinsic mCherry^negative^Tomm20^+^ mitochondria in GSCs; representative of at least 3 different optical fields with evidence of mitochondria transfer, across 3 experiments (per model). Animation of the 3D reconstructions can be seen in Supplementary Videos 1-2. (**B**) Z-stacks of L1 cells with internalized astrocyte-derived mito-mCherry^+^ mitochondria (yellow arrowheads) were utilized to perform linear colocalization analysis of mito-mCherry with Tomm20 (analyzed pixels denoted with horizontal white line in the “merged” channel micrograph. (**C**) Linear colocalization analysis from **Panel B** data. Magenta mito-mCherry peaks coincide with cyan Tomm20 peaks, indicating colocalization. Source data

Given our observation that astrocytes displayed high rates of mitochondria transfer to mouse models of GBM and their previously described interconnected nature with GBM in vivo^32^, we focused our subsequent studies on this glial cell population. We began by coculturing either mito-mCherry or mitoDsRed immortalized human astrocytes with six different human-derived GSCs (L1, DI318, 3832, GG16, P3 and BG5) for 3–4 d to interrogate the applicability of our findings in human models of disease. As with mouse GBM models, we found that human-derived GSCs acquired mitochondria from astrocytes, with flow cytometry revealing a transfer rate of 5–40% (Fig. 2c,d and Extended Data Fig. 4c). We confirmed the internalization of astrocyte mitochondria by confocal microscopy of the GSCs, whereby the fluorescent protein-tagged mitochondria were visualized entirely within GSCs with cytoplasm prelabeled with CellTrace dye or expressing GFP (Extended Data Fig. 4d,e). We confirmed that astrocyte-to-human GBM transfer of mitochondria was relevant in vivo using the human orthotopic GBM model (P3) derived from Fig. 1g. We identified mitoDsRed^+^ astrocytes (glial fibrillary acidic protein (GFAP^+^) and GFP^–^) surrounding GFP^+^ GBM cells with transferred mitoDsRed^+^ mitochondria (Fig. 2e and Extended Data Fig. 4f). ImageStream analysis on a distinct set of human-derived co-culture models, where donor astrocytes were tagged with mito-GFP and GBM recipients with red-fluorescent protein (RFP), revealed similar results with internalized donor mitochondria in recipient cells (Extended Data Fig. 4g–i). Finally, we confirmed that transferred mitochondria labeled with mito-mCherry expressed the mitochondrial protein translocator of outer mitochondrial membrane 20 (TOMM20) by colocalization analysis, and the protein was distributed within the mitochondrial network of the receiving GBM cell (Extended Data Fig. 5 and Supplementary Video 1 and 2). In summary, we observed mitochondria transfer from the TME to GBM in vivo and in vitro and identified astrocytes as a major mitochondria donor.

### GAP43^+^ MTs facilitate mitochondria transfer

**Fig. 3:**
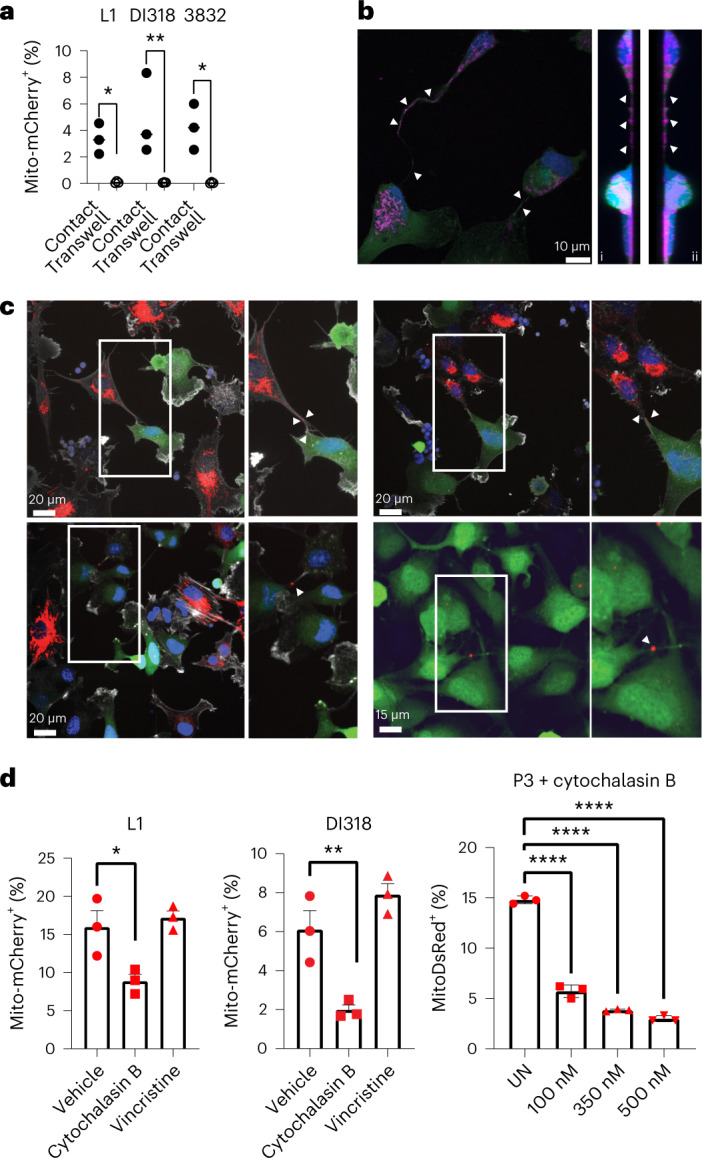
Astrocytes transfer mitochondria to GBM via actin-based intercellular connections. **a**, Human-derived GSCs (L1, DI318 and 3832; bottom) and astrocytes (top) were separated from each other with 5-μm porous transwell inserts. Twenty-four hours later, mitochondria transfer was analyzed based on mito-mCherry signal; *n* = 3 independent experiments; *P* = 0.02 (L1), 0.007 (DI318) and 0.01 (3832). Data were analyzed by two-way ANOVA. **b**, Presence of mitochondria within MTs connecting P3 GFP^+^ cells. Immunostaining with TOMM20, representative of at least eight ×100 images across three biologically independent co-cultures, is shown. Intratubular location is confirmed by *z* stacking, seen from the left in (i) and the right in (ii) side. Arrows indicate TOMM20^+^ signal. **c**, Co-culture between mitoDsRed^+^ astrocytes and GFP^+^ P3 cells immunostained with F-actin (white) to visualize membrane extensions connecting the two cell types, emphasized with high magnification. Images are representative of at least 20 ×60 images across three biologically independent co-cultures. Arrows indicate mitoDsRed^+^ mitochondria in intercellular connections and transferred mitoDsRed^+^ mitochondria. MitoDsRed^+^ mitochondria are also observed in MT connections between two tumor cells, confirming the exchange of mitochondria within the whole network of tumor–tumor/tumor–astrocyte connections. Insets: ×1.5 magnification. **d**, Human-derived GSCs were cocultured for 24 h with immortalized mito-mCherry^+^ and mitoDsRed^+^ human astrocytes in the presence of actin (cytochalasin B) or microtubule (vincristine) polymerization inhibitors or vehicle control (UN). The frequency of mito-mCherry^+^ or mitoDsRed^+^ GSCs was assessed by flow cytometry; *n* = 3 independent experiments. Data are presented as mean ± s.e.m. (L1 and DI318) and mean ± s.d. (P3); *P = 0.03 (L1), **P = 0.01 (DI318), ***P < 0.0001 (P3). Data were analyzed by one-way ANOVA with Holm–Sidak multiple comparison correction. Source data

**Extended Data Fig. 6:**
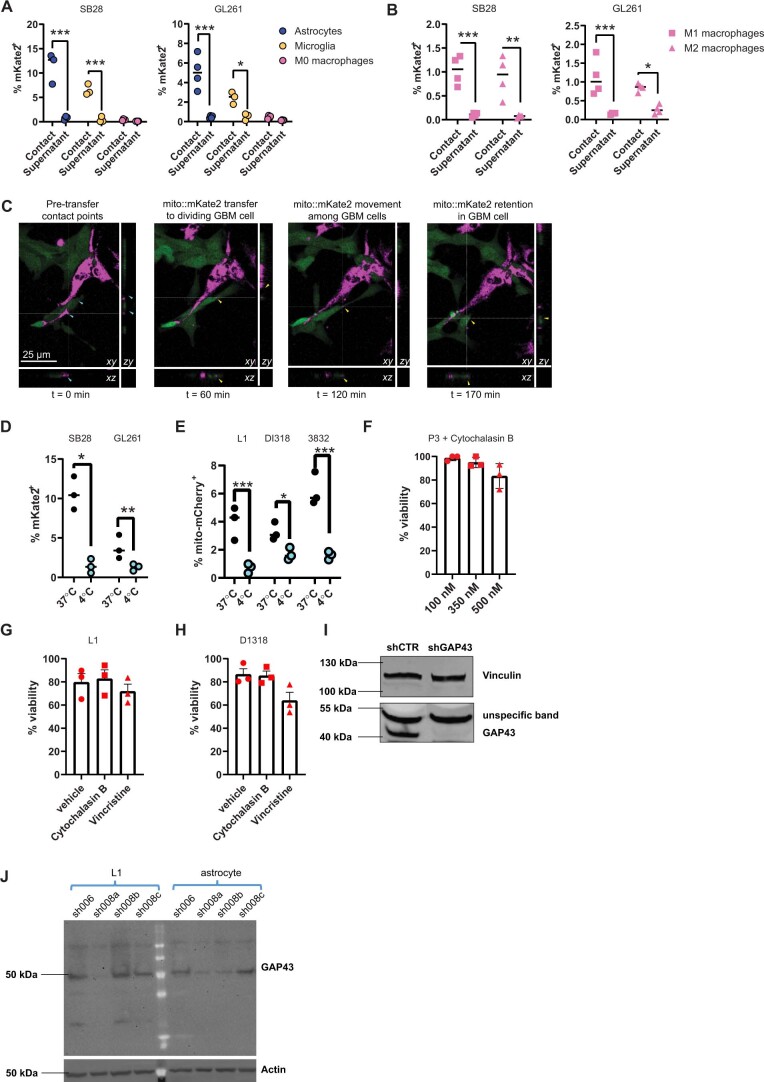
Transfer of host mitochondria to GBM cells is contact-, energy-, actin-, and GAP43-dependent: additional data. (**A-B**) GBM cells were co-cultured in direct contact with astrocytes, microglia, or M0/M1/M2 macrophages from mito::mKate2 mice for 2 hours (“contact”). Alternatively, culture supernatant conditioned by donor cells for 48 hours was transferred to GBM cell cultures (“supernatant”). Mitochondria transfer was analyzed by flow cytometry. n = SB28 (3 astrocytes, 3 microglia, 4 macrophages, 4 M1 macrophages, 4 M2 macrophages and GL261 (4 astrocytes, 3 microglia, 4 macrophages, 4 M1 macrophages, 4 M2 macrophages) independent experiments. * p = 0.03 (GL261 microglia), 0.02 (GL261 M2 macrophages), ** p < 0.02, *** p = <0.0001 (astrocytes), 0.001 (SB28 microglia), 0.0007 (SB28 M1 macropahges), 0.0004 (GL261 M1 macrophages), two-way ANOVA. (**C**) Confocal time-lapse images demonstrating real-time acquisition of astrocyte mitochondria (magenta) by SB28 cells (green). Cyan arrowheads point to contact points between SB28 cells and astrocytes. Yellow arrowheads point to transferred mito-mCherry^+^ astrocyte mitochondria inside SB28 cells. Video footage provided in Supplementary Video S3. (**D**) Mouse and (**E**) patient-derived GBM cells were co-cultured with mito::mKate2 or mito-mCherry (respectively) astrocytes for 2 hours at 37 °C or 4 °C. Graphs depict astrocyte mitochondria transfer to GBM cells. n = 3 independent experiments for each model. (**D**) * p = 0.04, ** p < 0.0003, (**E**) p = 0.0004 (L1), 0.03 (DI318), < 0.0001 (3832), two-way ANOVA with Sidak multiple comparison correction. (**F**) WST (viability) assay of P3 cells treated with cytochalasin B at indicated concentrations normalized to control. n = 3 independent experiments; mean ± SD. (**G-H**) Patient-derived GSCs were co-cultured for 24 h with mito-mCherry^+^ human astrocytes in the presence of actin (cytochalasin B) or microtubule (vincristine) polymerization inhibitors or vehicle control. Viability of GSCs was assessed by flow cytometry. n = 3 independent experiments; mean ± SEM. * p < 0.05, ** p < 0.01, ANOVA. (**I**) Knockdown of GAP43 in P3 GBM cells. Western blot for GAP43 in shGAP43 and shCTR P3 cells, representative of 2 independent experiments. Vinculin was used as a loading control. (**J**) Western blot of patient-derived GSCs (L1) and immortalized mito-mCherry human astrocytes, confirming knockdown of GAP43 in cell cultures (once) prior to use for experiment series in Fig. 4D (L1-sh008a and astrocytes-sh008b). Source data

We considered that mitochondria transfer could occur via secretion^14^ and/or cell contact^24,33^. To discriminate between the two mechanisms, we compared the mitochondria transfer rate of co-cultures to that of transferred donor-conditioned medium (mouse models; Extended Data Fig. 6a,b) or cells separated by a 5-μm porous transwell insert (human models; Fig. 3a). Regardless of the separation method, transfer primarily occurred when donor and recipient cells were in physical contact, and there was only low-level transfer by secretion that was near the limit of detection of our assays. Live confocal microscopy of mitochondria transfer from astrocytes to mouse GBM cells was also visualized in adjacent cells (Extended Data Fig. 6c and Supplementary Video 3). Transfer was abrogated in both mouse and human models when co-cultures were incubated at 4 °C (Extended Data Fig. 6d,e), suggesting that this was an active, energy-dependent process rather than a passive event. Taken together, these results indicate that active physical interaction of tumor and donor cells is required for effective mitochondria transfer, in line with imaging of in vivo brain tumor models (Figs. 1 and 2 and Extended Data Figs. 1 and 2).

MTs are established conduits of intercellular communication and network formation in GBM and, thus, are also potential mediators of mitochondria transfer^24,32,34–36^. To visualize mitochondria within the interfaces of GBM cells, we performed confocal microscopy of co-cultures immunostained for TOMM20 and actin. In addition to a perinuclear localization, mitochondria were found in MT connections between tumor cells (P3 model; Fig. 3b), consistent with a previous study reporting that mitochondrial components localize in between astrocyte and GBM cell connections in vitro^37^. Their intratubular location was confirmed by *z* stack. Importantly, confocal microscopy of mitoDsRed^+^ astrocytes and GFP^+^ P3 co-cultures immunostained with actin showed MTs with mitoDsRed^+^ mitochondria connecting both cell types. These were present in the vicinity of transferred mitochondria (Fig. 3c). We also found astrocytic mitoDsRed^+^ donor mitochondria shuttling between connected tumor acceptor cells, indicating that astrocytic mitochondria can be further exchanged within the complete network of tumor–tumor connections (Fig. 3c). After visualizing mitochondria in transit along intercellular connections containing actin filaments, we hypothesized that the actin cytoskeleton was critical in facilitating mitochondria transfer. Indeed, inhibition of actin polymerization by cytochalasin B resulted in a significant decrease in transfer rate, without an effect on cell viability (Fig. 3d and Extended Data Fig. 6f–h). By contrast, inhibition of tubulin polymerization with vincristine had no effect on mitochondria transfer (Fig. 3d).

**Fig. 4:**
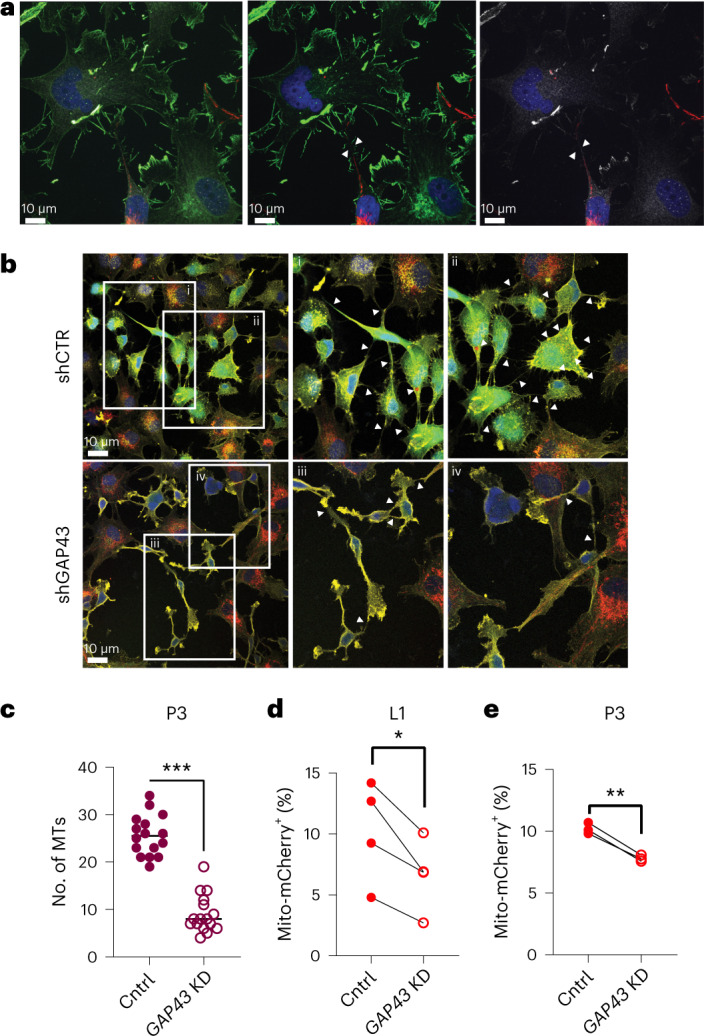
GAP43 facilitates mitochondria transfer via tumor–astrocyte MTs. **a**, Presence of GAP43^+^ MT-like connections (arrows) between mitoDsRed^+^ astrocytes and P3 tumor cells. Immunostaining for GAP43 (white) and actin (green) is shown. Left: mitoDsRed (red), GAP43 and actin. Middle: mitoDsRed and actin. Right: mitoDsRed and GAP43. Images are representative of at least four ×100 images across three biologically independent co-cultures. **b**, Co-culture of mitoDsRed^+^ astrocytes and GFP^+^ P3 short hairpin GAP43 (shGAP43) cells indicates fewer membrane connections between donor and recipient cells than observed in GFP^+^ P3 short hairpin control (shCTR) cells. Immunofluorescence staining with F-actin (yellow) is shown and is further visualized at increased magnification (i–iv). Arrows indicate MTs. Insets: 2 × magnification. **c**, Quantification of MTs from **b** across 16 independent ×60 images across *n* = 4 independent co-culture experiments per group. Data are shown as the mean ± s.d.; ***P < 0.0001. Data were analyzed by two-tailed *t*-test; Cntrl, wild type; KD, knockdown. **d**, Wild-type or *GAP43*-knockdown L1 human-derived GSCs were cocultured for 24 h with matching wild-type or *GAP43*-knockdown mito-mCherry^+^ astrocytes. Astrocyte-derived mitochondria transfer to GSCs was quantified by flow cytometry; *n* = 4 independent experiments; *P = 0.01. Data were analyzed by two-tailed *t*-test. **e**, Wild-type or *GAP43*-knockdown P3 human-derived GSCs were cocultured for 24 h with mitoDsRed human astrocytes. Astrocyte-derived mitochondria transfer to GSCs was quantified by flow cytometry; *n* = 3 independent experiments; **P = 0.001. Data were analyzed by two-tailed *t*-test. Source data

Previous work showed that GAP43 facilitated the formation of an interconnected network of GBM cells in vivo, which enabled connexin 43-mediated propagation of calcium waves^24^. GAP43 is an actin-associated protein that facilitates neurite outgrowth via growth cones^38,39^. We visualized that GAP43 also localized to the cellular projections of GBM cells and astrocytes in co-culture (Fig. 4a). When we knocked down the expression of GAP43 in human-derived GSCs, we observed a decrease in the number of cellular projections (Fig. 4b,c and Extended Data Fig. 6i), as previously reported^24,34^. Importantly, we found that mitochondria transfer from astrocytes was significantly decreased when GAP43 was knocked down in GSCs alone or in both GSCs and astrocytes (Fig. 4d,e and Extended Data Fig. 6i,j). These data identify a role for GAP43 in facilitating mitochondria transfer from astrocytes to GBM.

### Astrocyte mitochondria metabolically reprogram GBM cells

**Extended Data Fig. 7:**
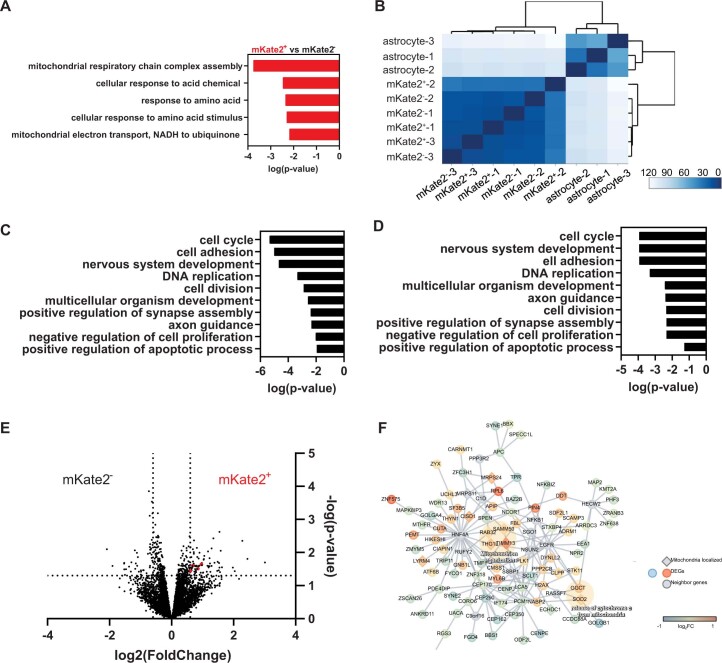
Mitochondria transfer alters expression of metabolism gene pathways in recipient GBM cells. (**A-F**) RNAseq analysis from n = 3 independent co-culture experiments. (**A**) Top 5 pathways enriched in RNAseq data from sorted mKate2^+^ versus mKate2^−^SB28 cells based on genes upregulated >1.5 fold with p < 0.05 (one-tailed hypergeometric enrichment test, unadjusted p-values). (**B-D**) Data supporting purity of sorted cell populations in RNAseq experiments, given distinct transcriptomic signature of sorted astrocytes vs. GBM cells. (**B**) Heatmap demonstrating separate unsupervised hierarchical clustering of astrocytes from mKate2^−^ and mKate2^+^ tumor cells based on gene expression profiling. GO pathway analysis of differentially expressed genes between (**C**) mKate2^−^ cells and astrocytes and (**D**) mKate2+ cells and astrocytes. One-tailed hypergeometric enrichment test, unadjusted p-values. (**E**) Volcano plot representing differential gene expression signature of mKate2^+^ versus mKate2^−^ cells. Dashed lines mark fold change > 1.5 and p-value < 0.05. Negative binomial distribution DESeq2 testing, unadjusted p-value. Genes that are mapped to mitochondria-related networks are shown in red. (**F**) Differentially up-regulated genes are enriched in the mitochondrial network. Protein-protein interaction network for the differentially expressed genes. Mouse genes were mapped to human genes according to NCBI HomoloGene. Protein-protein interactions were extracted for these genes/proteins using our human protein interactome. Diamond-shaped nodes indicate that these genes are mitochondrially localized based on the Human MitoCarta2.0 database. Node color shows the log2 fold change of the genes. Source data

We hypothesized that receiving entire organelles from astrocytes would have biologically important downstream functional sequelae in recipient GBM cells. We first interrogated how mitochondria transfer affected cells transcriptionally by bulk RNA sequencing (RNA-seq) of sorted mouse GBM cells with (mKate2^+^) or without (mKate2^−^) astrocyte mitochondria acquisition from co-cultures (Extended Data Fig. 7a). Unsupervised clustering of RNA-seq data revealed that tumor cells had a distinct gene expression profile compared to astrocytes, confirming that sorted mKate2^+^ SB28 cells were not meaningfully contaminated by astrocytes from the co-culture (Extended Data Fig. 7b–d). Genes that were upregulated >1.5-fold in mKate2^+^ tumor cells compared to mKate2^–^ tumor cells (Extended Data Fig. 7e,f and Supplementary Table 1) were enriched within pathways related to mitochondrial biology, in particular electron transport and mitochondrial organization (Extended Data Fig. 7a).

**Fig. 5:**
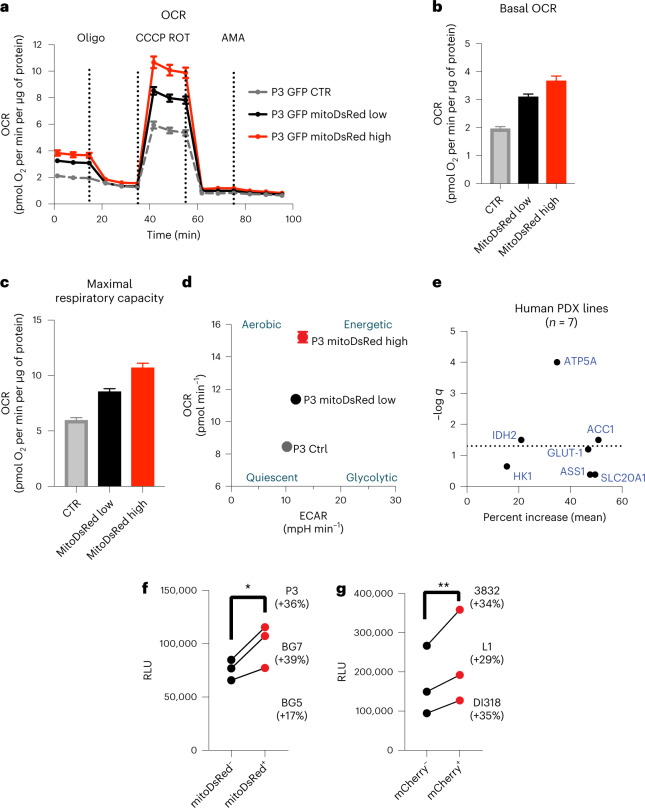
Acquisition of astrocyte mitochondria enhances ATP production by mitochondrial respiration in recipient GBM cells. **a**, Oxygen consumption rate (OCR) was measured in GFP^+^ P3 cells sorted after transfer with a high or low amount of mitoDsRed^+^ mitochondria from donor mitoDsRed^+^ astrocytes and was compared with that in sorted GFP^+^mitoDsRed^–^ P3 cells from the same co-culture. Following readings of basal respiration, the stepwise addition of 3 mM oligomycin (Oligo) to measure leak respiration, 1.5 mM CCCP to quantify maximal and reserve capacity and 1 mM rotenone (ROT) followed by 1 mM antimycin A (AMA) to measure non-mitochondrial respiration was performed; CTR, control. **b**, Basal oxygen consumption gradually increased with cumulative mitochondrial content in GFP^+^ P3 cells. **c**, Maximal respiratory capacity gradually increased with cumulative mitochondria content in GFP^+^ P3 cells. **d**, Energy map indicating that GFP^+^ P3 cells with a higher degree of mitochondria transfer from astrocytes have a more aerobic and energetic phenotype than GFP^+^ P3 cells with less and no mitochondria transfer. Data in **a**–**d** are from a representative experiment from a total of three independent experiments. Data shown as mean ± s.e.m.; *n* = 18 (control), 22 (mitoDsRed low) and 9 (mitoDsRed high) technical replicates; statistical comparison of technical replicates is not shown. ECAR, extracellular acidification rate; mpH, milli pH. **e**, Seven distinct human-derived GSCs were cocultured for 4 d with immortalized mito-mCherry^+^ human astrocytes and stained with antibodies recognizing key metabolic proteins for downstream flow cytometry quantification. Protein expression was compared between mito-mCherry^+^ and mito-mCherry^−^ cells by mixed-effects model analysis. The dotted line represents the statistical significance threshold (false-discovery rate (FDR) < 0.05). **f**, ATP levels in sorted mitoDsRed^+^ versus mitoDsRed^–^ cells from distinct human-derived GSCs, measured with the CellTiter-Glo luminescence assay; *n* = 3 independent experiments; *P = 0.04. Data were analyzed by two-tailed ratio paired *t*-test; RLU, relative light units. **g**, ATP levels in sorted mito-mCherry^+^ versus mito-mCherry^−^ cells from three distinct human-derived GSC lines, assessed by CellTiter-Glo luminescence assay; *n* = 3 independent experiments; **P = 0.003. Data were analyzed by two-tailed *t*-test. Source data

**Extended Data Fig. 8:**
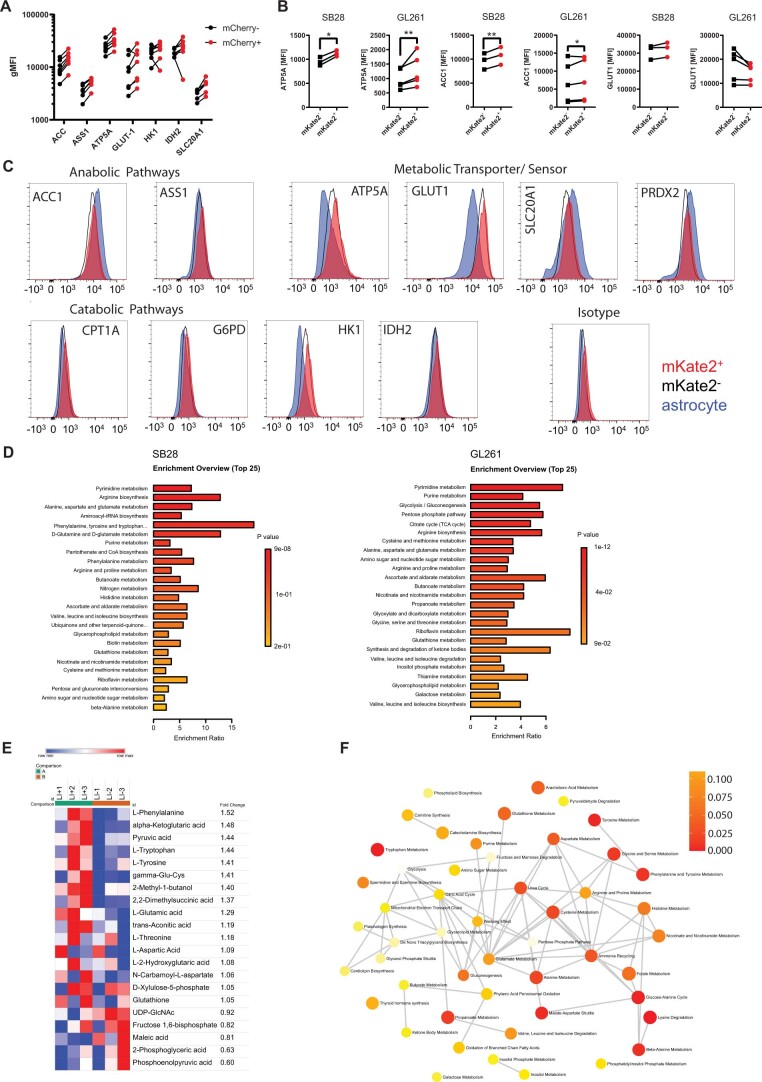
Mitochondria transfer alters GBM metabolism at protein and metabolomic levels. (**A**) Aggregate data that is summarized in Fig. 5E. Graph depicts gMFI of metabolic protein expression assessed by flow cytometry in seven distinct patient-derived GSCs co-cultured with immortalized human mito-mCherry astrocytes for 4 days. Results of statistical analysis by mixed-effects model is summarized in Fig. 5E. (**B**) Mouse GBM cell lines were co-cultured with mito::mKate2^+^ astrocytes for 24 h and stained with antibodies against key metabolic proteins, as denoted. Expression levels were assessed by flow cytometry. Aggregate data from n = 3 (SB28) and 5 (GL261) independent experiments. Panels depict isotype background-corrected, geometric mean fluorescence intensity (gMFI) of the expression of the indicated metabolic proteins. * p = 0.02 (SB28), 0.03 (GL261), ** p = 0.004 (SB28), 0.009 (GL261), paired 2^−^tailed t-test. (**C**) Representative histograms of data summarized in **Panel B**, depicting differential expression of critical metabolic proteins by astrocytes and mKate2^+^ and mKate2^-^ SB28 cells. Astrocytes had higher levels of acetyl-CoA carboxylase (ACC1), SLC20A1 and peroxiredoxin-2 (PRDX2), indicative of more oxidative phosphorylation. In contrast, tumor cells had higher levels of glucose transporter 1 (GLUT 1) and hexokinase 1 (HK1), pointing to a more glycolytic metabolism. (**D**) Mouse GBM cells were sorted from co-cultures with mouse mito::mKate2 astrocytes and analyzed by targeted metabolomics. Analysis depicts metabolic pathways upregulated in mito::mKate2^+^ vs. mito::mKate2^-^ GBM cells (MetaboAnalyst 5.0 one-tailed hypergeometric test; unadjusted p-values). This data was used in the combined analysis identifying mutually upregulated pathways in both models, shown in Fig. 6A. (**E**) L1 cells were co-cultured with immortalized human mito-mCherry astrocytes for 4 days and sorted for metabolite analysis. Heat map showing comparison of abundance of metabolites in mito-mCherry^+^ L1 cells (L1 + ) when compared to mito-mCherry^−^ L1 cells (L1-). (**F**) Network of metabolites enriched more in mito-mCherry^+^ L1 cells when compared to mito-mCherry^−^ L1 cells (MetaboAnalyst 5.0 one-tailed hypergeometric test; unadjusted p-values). Source data

Given the central role of mitochondria in ATP production and the results of our RNA-seq analysis, we investigated whether an increase in functional mitochondria through transfer causes a measurable change in metabolic parameters, in particular oxygen consumption rate. We performed a mitochondrial stress test on GFP^+^ acceptor cells (P3 model) with high and low mitochondria transfer from mitoDsRed^+^ astrocytes compared to control cells from the same co-cultures using the Seahorse system. After measuring the basal respiration rate, we sequentially added electron transport chain inhibitors to evaluate the changes in maximal respiration capacity. Interestingly, both basal respiration and maximal respiration rate were increased in accordance with mitochondria transfer (Fig. 5a–c). When dividing the cells into metabolic subgroups based on basal respiratory and glycolytic rates, cells with the highest mitochondria transfer were more aerobic and energetic than cells with lower transfer and controls (Fig. 5d). We further characterized the metabolic profile of a panel of GSCs after co-culture with human astrocytes by adapting a previously reported metabolic flow cytometry panel^40^. The mitochondrial ATP synthase subunit ATP5A was among the most consistently upregulated metabolic proteins in recipient GSCs (Fig. 5e and Extended Data Fig. 8a) and in mouse models of GBM (Extended Data Fig. 8b,c). We evaluated whether these cells with higher respiratory capacity and ATP synthase levels generated more ATP. Across multiple human-derived GBM models, we verified that GBM cells that acquired mitochondria from astrocytes also had higher levels of ATP as measured by luminescence reporter assay (Fig. 5f,g). Collectively, these data show that transferred astrocyte mitochondria are bioactive and lead to augmented mitochondrial respiration and ATP production in recipient GBM cells.

**Fig. 6:**
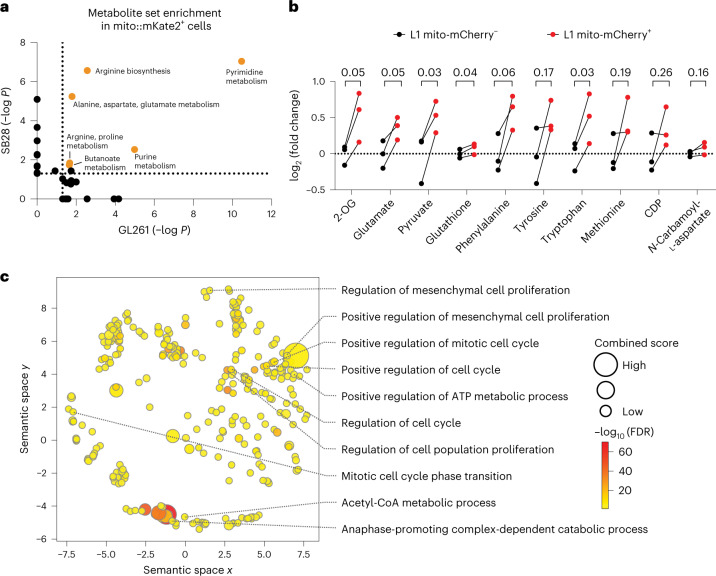
Mitochondria transfer from astrocytes reprograms GBM metabolism. **a**, Metabolic pathway analysis (MetaboAnalyst) of metabolites enriched by >20% in mito::mKate2^+^ versus mito:^:^mKate2^–^ mouse GBM cells. For each cell model, *n* = 3 independent co-culture experiments were pooled. Dotted lines represent the cutoff for statistical significance (*P* < 0.05). Pathways significantly enriched in both GBM models are highlighted with orange and are labeled with the pathway name. *P* values were calculated with the MetaboAnalyst 5.0 web tool using the one-tailed hypergeometric test for enrichment analysis (expected versus observed metabolites enriched in each pathway). **b**, L1 cells were cocultured with immortalized mito-mCherry^+^ human astrocytes for 4 d. Relative abundances of metabolites in mito-mCherry^+^ versus mito-mCherry^−^ L1 cells indicate higher amino acid and glutathione metabolism in mito-mCherry^+^ L1 cells; *n* = 3 independent co-culture experiments. Data were analyzed by paired two-tailed *t*-test; 2-OG, α-ketoglutarate; CDP, cytidine-5'-diphosphate. **c**, Phospho-array pathway analysis (Enrichr) of protein phosphorylation sites upregulated in mito-mCherry^+^ versus mito-mCherry^−^ L1 cells, depicted with dimensionality reduction in semantic *xy* space. Dots represent significantly upregulated pathways (FDR < 0.05). Selected pathways associated with cell metabolism and proliferation are labeled; *n* = 3 independent co-culture experiments were pooled and analyzed. Source data

Mitochondria can further influence cellular biology by modulating diverse metabolic pathways^41^, and there is increasing evidence that they influence intracellular phosphorylation signaling cascades^42,43^. We thus performed a metabolite mass spectrometry assay and 584-site protein phosphorylation array on sorted GBM cells from co-cultures with astrocytes to interrogate the functional consequence of mitochondria uptake. Metabolite enrichment analysis revealed multiple upregulated metabolic pathways in mKate2^+^ GBM cells shared between both GL261 and SB28 mouse models (Fig. 6a, Extended Data Fig. 8d and Supplementary Table 2). Among these pathways was amino acid and nucleotide metabolism, previously linked to proliferation, self-renewal and tumorigenicity in GBM^44–46^. Metabolomic analysis of the sorted human-derived L1 cells showed that mCherry^+^ cells have higher amounts of glutamate, α-ketoglutarate, glutathione and essential amino acids than mCherry^−^ cells (Fig. 6b and Extended Data Fig. 8e,f). Glutamate is one of three amino acids in glutathione, a major cellular antioxidant, and glutamate metabolism is a principal route for assimilation of nitrogen in de novo nucleotide synthesis, which promotes numerous oncogenic processes in gliomas^47,48^. This suggests that transfer of mitochondria may be helping the GBM cells support proliferation and protect against oxidative stress. Our phosphoprotein array also revealed changes in the phosphorylation levels of numerous signaling and effector proteins in human-derived GSCs that received astrocyte mitochondria, many of which mapped to proliferation and cell cycle pathways (Fig. 6c and Supplementary Table 3). These findings are consistent with metabolic reprogramming of recipient cells beyond increased mitochondrial respiration, along with intracellular signaling with potential effects on cell cycle regulation and other processes.

### Mitochondria transfer increases GBM tumorigenicity

**Fig. 7:**
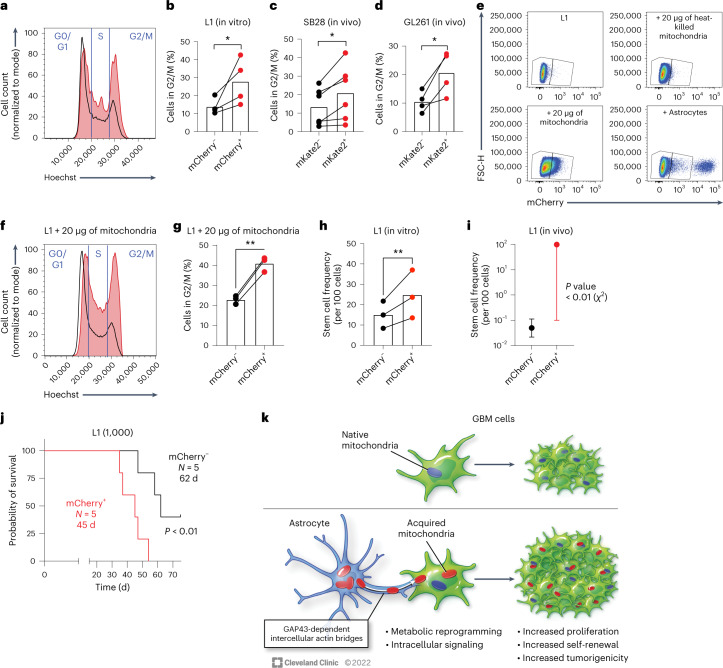
Mitochondria transfer from astrocytes drives GBM cell proliferation, self-renewal and tumorigenicity. **a**,**b**, Human-derived GSCs (L1) were cocultured with immortalized mito-mCherry^+^ human astrocytes. Representative histograms (**a**) and aggregate data (**b**) of *n* = 4 independent experiments depicting cell cycle analysis by flow cytometric DNA quantification in GSCs that acquired astrocyte mitochondria (mCherry^+^, red histogram/dots) versus those that did not (mCherry^–^, black histogram/dots) are shown; *P = 0.04. Data were analyzed by two-tailed *t*-test. **c**,**d**, Cell cycle analysis by ex vivo flow cytometry DNA quantification in GFP-expressing mouse GBM cells obtained from orthotopic tumors in mito::mKate2 mice; *n* = 4–6 mice per tumor model and *N* = 6 (SB28; **c**) and 4 (GL261; **d**) mice per group; *P = 0.02 (SB28) and 0.03 (GL261). Data were analyzed by two-tailed paired *t*-test. **e**–**g**, Cell-free mito-mCherry^+^ astrocyte mitochondria (intact or heat killed) were added to an L1 culture; vehicle (PBS) or live mito-mCherry^+^ astrocytes were added to control wells. **e**, Representative dot plots depicting the identification of L1 cells that acquired cell-free mitochondria or mitochondria from cocultured astrocytes. In the +astrocyte condition, the mCherry^hi^ population outside of the gates is composed of the mito-mCherry^+^ astrocytes. Representative histograms (**f**) and aggregate data (**g**) depicting cell cycle analysis of GSCs that acquired cell-free astrocyte mitochondria (mCherry^+^, red histogram/dots) versus those that did not (mCherry^–^, black histogram/dots) are shown; *n* = 3 independent experiments; **P = 0.003. Data were analyzed by two-tailed paired *t*-test. **h**,**i**, Estimated stem cell frequency in mCherry^+^ versus mCherry^–^ human-derived GBM models sorted from astrocyte co-cultures and subjected to in vitro limiting dilution sphere formation assay (**h**) or in vivo orthotopic tumor initiation assay (**i**); **P = 0.002, ratio paired *t*-test, *n* = 3 independent experiments (**h**); *P* = 0.005; compiled data from *n* = 15 NOD *scid* gamma (NSG) mice per group (distributed across three cell-dose levels). Data are shown as mean ± 95% confidence interval; *χ*^2^ test with 1 degree of freedom analyzed by Extreme Limiting Dilution Analysis (ELDA; **i**). **j**, Survival of mice injected orthotopically with 1,000 sorted L1 GSCs per animal; *P* = 0.009. Data were analyzed by log-rank test. Survival analysis of other dose levels is presented in Extended Data Fig. 8. **k,** Schematic overview of findings. Source data

**Extended Data Fig. 9:**
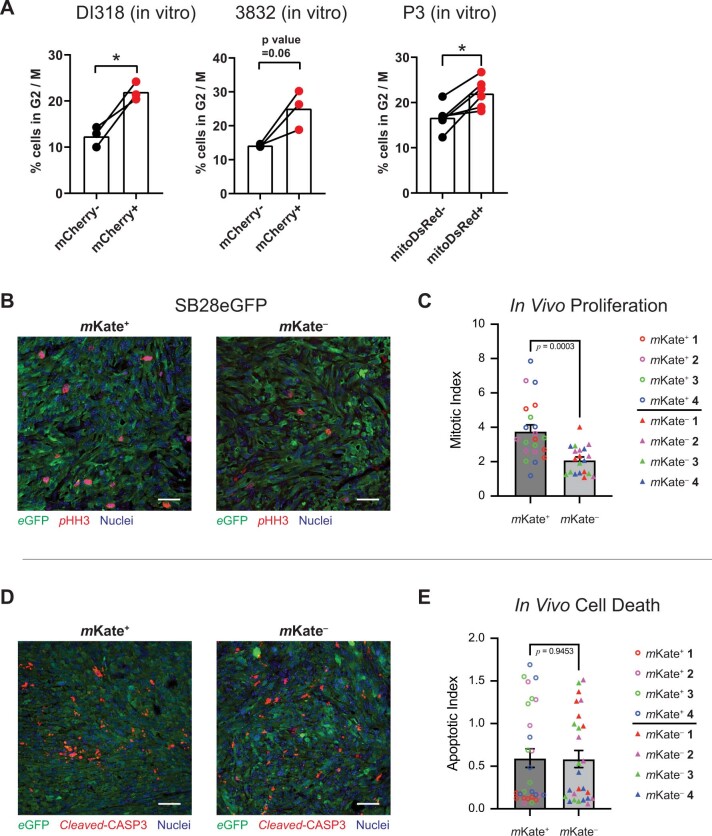
GBM cells that acquire astrocyte mitochondria are more proliferative. (**A**) Three additional patient-derived GSC specimens were co-cultured with immortalized human mito-mCherry or mitoDsRed astrocytes for cell cycle analysis by flow cytometry DNA quantification. GBM cells that acquired astrocyte mitochondria (mCherry^+^ or mitoDsRed^+^) were more likely to be in G2/M phase. N = 3 (DI318), 3 (3832) and 6 (P3) biologically independent samples. * p = 0.04 (DI318), 0.01 (P3), 2-tailed t-test. (**B-E**) mKate2+ and mKate2- SB28 cells were sorted from co-cultures with mito::mKate2 astrocytes and implanted orthotopically in C57BL/6 mice. At humane endpoint, animals were euthanized and their brains fixed for subsequent histologic analysis. (**B**) Representative micrographs and (**C**) aggregate data from n = 4 mice per group assessing tumor mitotic index by phospho-histone H3 (pHH3) staining. p = 0.0003. (**D**) Representative micrographs and (**E**) aggregate data from n = 4 mice per group assessing apoptotic cell death of GBM cells by cleaved caspase-3 (cleaved-CASP3) staining. p = 0.9. Scale bar denotes 25 µm. Mean ± SEM, 2-tailed t-test. Source data

To determine whether the metabolic and signaling changes that we observed in GBM cells that acquired astrocyte mitochondria resulted in altered cell cycle regulation, we performed cell cycle analysis in our models of mitochondria transfer by DNA staining and flow cytometry across multiple human-derived GSC models. We observed a consistent increase in the proportion of cells in the proliferative G2/M phases of the cell cycle following acquisition of astrocyte mitochondria (Fig. 7a,b and Extended Data Fig. 9a). This observation was consistent in vivo when analyzing mouse GBM models from orthotopic tumors established in mito::mKate2 mice (Fig. 7c,d). Moreover, orthotopic mouse GBM tumors originating from sorted cells that had acquired astrocyte mitochondria had a higher mitotic index (Extended Data Fig. 9b–e), suggesting that the increased proliferation phenotype was retained in vivo. Previous reports demonstrated that GSCs could take up isolated cell-free mitochondria added to cell culture medium^49^. Addition of astrocyte-derived mitochondria to human-derived GSCs was sufficient to recapitulate the increased proportion of cells in G2/M phases of the cell cycle (Fig. 7e–g). These data suggest that the transfer of astrocyte mitochondria impacts cell cycle regulation in human GBM models.

**Extended Data Fig. 10:**
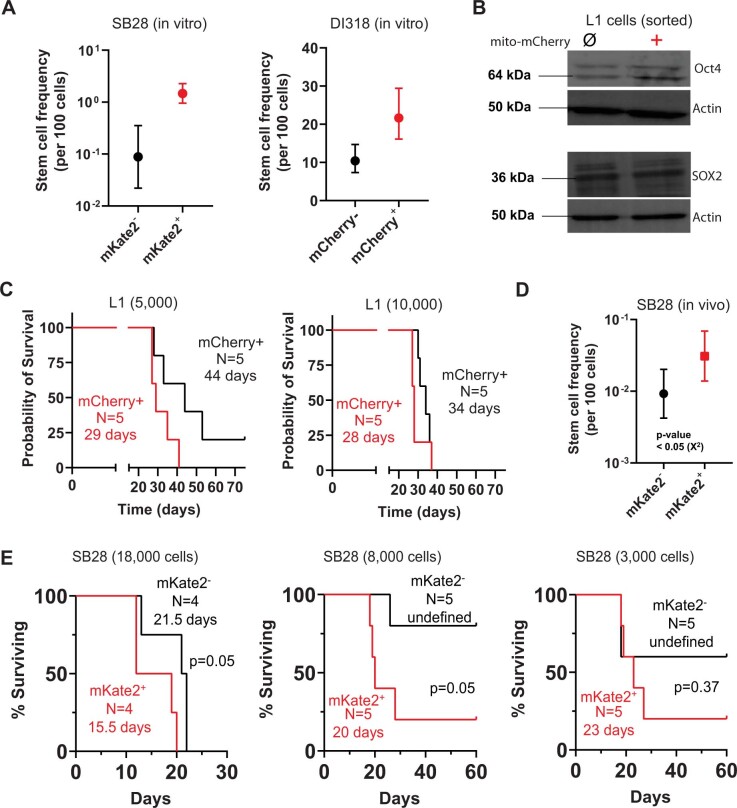
Acquisition of astrocyte mitochondria enhances self-renewal and tumorigenicity in mouse and human models of disease. (**A**) Mouse GBM cells (SB28) and additional patient-derived GBM cells (DI318) were sorted from co-cultures and assayed for self-renewal by in vitro limiting-dilution sphere-formation assay. Representative experiments from 3 (SB28) and 2 (DI318) independent experiments are shown. Mean ± 95% confidence interval; analyzed by ELDA; p-value of individual experiments not shown. (**B**) mito-mCherry^+^ and mito-mCherry^−^patient-derived GSCs (L1) sorted from one of the astrocyte co-cultures were analyzed by western blotting of stem cell transcription factors Oct4 and SOX2. (**C**) Additional survival curves of animals implanted with sorted L1 cells from co-cultures for the *in vivo* orthotopic tumor-initiation assay summarized in Fig. 7I. Log-rank test. (**D**) *In vivo* orthotopic tumor-initiation assay using mouse SB28 GBM cells sorted from mito::mKate2 astrocyte co-cultures. n = 14 mice per group (total 28 mice). Mean ± 95% confidence interval. p = 0.03, χ^2^ test with 1 degree of freedom, analyzed by ELDA analysis. (**E**) Animal survival and tumor penetrance data at 3 different cell doses; used to calculate tumor initiating cell frequency in **Panel D**. Log-rank test. Source data

To further assess the impact of mitochondria acquisition on self-renewal, we performed limiting dilution sphere formation assays using sorted GBM cells with and without astrocyte mitochondria. In both human and mouse GBM models, we found that cells that acquired astrocyte mitochondria had significantly higher self-renewal capacity, represented as a higher estimated stem cell frequency (Fig. 7h and Extended Data Fig. 10a). While human-derived GSC models expressed high levels of the pluripotency transcription factor SOX2, we found that GSCs that acquired astrocyte mitochondria further upregulated another self-renewal transcription factor, OCT4 (Extended Data Fig. 10b). These findings suggest that mitochondria transfer from astrocytes consists of a previously undescribed TME interaction that promotes GBM self-renewal.

Increased proliferative capacity and self-renewal are central hallmarks of cancer; we thus hypothesized that mitochondria transfer from astrocytes promotes tumorigenicity of GBM. We tested this hypothesis by assessing the lethality (time to death or humane endpoint) and tumor initiation capacity of human-derived GBM cells with and without acquisition of human astrocyte mitochondria by orthotopic implantation in immunocompromised mice. These studies revealed that tumors led to symptomatic or lethal disease much faster when originating from GSCs that had acquired mitochondria from astrocytes and had a significantly higher in vivo tumor initiation capacity (Fig. 7i,j and Extended Data Fig. 10c). This observation was similar in mouse GBM models (Extended Data Fig. 10d,e). Thus, beyond altering the phenotype of GBM cells in vitro, mitochondria transfer from astrocytes increases the in vivo tumorigenicity of these cells in animal models.

## Discussion

Organelle transfer is an increasingly recognized biological process in models of GBM^35–37^ and other cancers^50–52^. Most of the existing knowledge on mitochondria transfer in cancer relies on depicting mitochondrial exchange among tumor cells, in purely in vitro systems, and/or using tumor cells artificially depleted of mitochondria. Thus, the role of mitochondria transfer from the TME to cancer cells (and to GBM in particular) remains poorly understood. Specifically, there is a lack of understanding about the in vivo relevance of mitochondria transfer from the GBM microenvironment, the cell types involved, the mechanism of transfer and its downstream effects on cellular function and tumorigenicity in disease-relevant contexts.

We found that in the context of GBM, mitochondria transfer from the TME is a frequent in vivo event and involves brain-resident glial cell donors. Here, we focused mechanistically on astrocytes as donor cells given their abundance in the brain, and our data identify them as important mitochondrial donors. Our data support that astrocytes in the TME form physical actin-based connections with GBM cells that have strong similarity to the previously described MTs, which are network-forming connections between GBM cells. Our data also support an extension of the previously described interconnected network of GBM to non-malignant astrocytes that has recently been verified by exchange of calcium waves within this network in vivo^24,32^. GAP43, typically found in neuronal projections and critical to GBM growth and the tumor cell network^24^, mediates mitochondria transfer from astrocytes in a previously undescribed role for this actin-associated protein.

Our findings further demonstrate that mitochondria transfer from astrocytes to GBM cells drives metabolic reprogramming toward oxidative respiration with increased ATP production. Mitochondria transfer also leads to intracellular signaling via protein phosphorylation linked to cellular proliferation and cell cycle progression.

The central finding of our study is that mitochondria transfer from a non-malignant cell of the TME promotes a highly tumorigenic cell phenotype characterized by both increased proliferative capacity and self-renewal (Fig. 7k). This manifested as higher penetrance and faster lethality of orthotopic tumors in vivo. Our findings suggest that the phenotype of increased proliferation and self-renewal driven by acquisition of astrocyte mitochondria before intracranial tumor implantation is sufficient to lead to increased tumorigenicity, while lacking this phenotype at the time of experimental tumor initiation leads to lower GBM cell proliferation. Thus, mitochondria transfer and the shift to oxidative metabolism comprises a fundamental protumorigenic interaction of GBM with its microenvironment. Our study adds a mechanistic understanding to this understudied process, forming the basis of future studies, which could also have broad applicability to tumors outside the central nervous system and other pathological contexts.

## Methods

### Ethics statements

Human material was obtained from surgeries performed at the Haukeland University Hospital (Bergen, Norway). Written consent was obtained from individuals with procedures that were approved for the projects (project numbers 013.09 and 151825) by the Regional Ethical Committee. Animal experiments were approved by the Institutional Animal Care and Use Committee of Cleveland Clinic and local ethical committee. Animals were treated in accordance with the Norwegian Animal Act.

### Human cell culture

The GSC lines P3, GG16 (provided by F. Kruyt, University of Groningen), BG5 and BG7 (ref. ^15^) were all derived from *IDH* wild-type biopsy specimens from individuals with GBM; 3832 cells were provided by J. Rich (University of Pittsburgh Medical Center) and have been described previously^53^. L1 cells were obtained from B. Reynolds (University of Florida) and have been described previously^54^. DI318 cells were obtained from the Rose Ella Burkhardt Brain Tumor Center biorepository and have been described previously (Cleveland Clinic)^55^.

Human-derived GBM cells were cultured in ‘complete’ Neurobasal medium (NBM): Neurobasal without phenol red (Gibco), supplemented with 2% B-27 supplement (Gibco), 1 mM sodium pyruvate (Gibco), 2 mM l-glutamine (Gibco), 1 U ml^–1^ penicillin + 1 μg ml^–1^ streptomycin (Cleveland Clinic Media Preparation Core), 20 ng ml^–1^ epidermal growth factor (EGF) and 20 ng ml^–1^ fibroblast growth factor 2 (FGF-2; R&D Systems). EGF was not added to P3 cells. Immortalized normal human astrocytes (provided by R. Pieper at University of California, San Francisco, and P. Øyvind Enger at Universitetet i Bergen) were cultured in tissue culture vessels (adherent) in neural stem cell (NSC) medium (DMEM-F12 (Media Preparation Core), 1 U ml^–1^ penicillin + 1 μg ml^–1^ streptomycin, 5% fetal bovine serum (FBS), 1% N2 supplement (Thermo Fisher Scientific), 20 ng ml^–1^ EGF and 20 ng ml^–1^ FGF-2 (R&D Systems)). For experiments involving downstream in vitro limiting dilution assays with L1 and DI318 cell lines, human-derived cells were maintained in DMEM (Cleveland Clinic Media Preparation Core) supplemented with 10% FBS (Thermo Fisher), 1 U ml^–1^ penicillin and 1 μg ml^–1^ streptomycin (Cleveland Clinic Media Preparation Core) for at least 7 d before co-culture with astrocytes.

Human-derived xenograft D456 was provided by D. Bigner (Duke University), and the JX22 cell line was provided by J. Sarkaria (Mayo Clinic). DMEM-F12 containing 10 ng ml^–1^ EGF, 10 ng ml^–1^ FGF, 1% sodium pyruvate, 2% GEM21 (Gemini Bio) and 1 U ml^–1^ penicillin + 1 μg ml^–1^ streptomycin was used to culture the human-derived xenograft lines.

### Lentiviral transductions of human cells

Human astrocytes were transduced with mito-mCherry lentivirus (Takara) and selected by fluorescence-activated cell sorting. GAP43 short hairpin RNA (shRNA) and non-target (control) lentiviruses were generated in-house according to the protocol described by Tisconia et al.^56^. shRNA sequences and sources are described in Supplementary Table 4.

Lentiviral vectors encoding mitoDsRed (Addgene, 44386), enhanced GFP (eGFP)^57^, mito-GFP (Addgene, 44385) and shRNA against *GAP43* (ref. ^24^) were prepared and titrated according to a protocol reported previously^58^. The mitochondria donor cells were transduced with pLV-mitoDsRed. The acceptor cells were transduced with a lentiviral eGFP vector^57^ or, for indicated in vivo experiments, with pLV-mito-GFP.

### Human cell co-culture mitochondria transfer assessment by flow cytometry

L1, DI318 and 3832 cells were cultured in tissue culture vessels (adherent) precoated with Geltrex (Thermo Fisher Scientific; 1:250 in serum-free medium overnight) in 90% complete Neurobasal + 10% NSC medium for 4 d, unless otherwise indicated. For *GAP43*-knockdown experiments, co-culture time was reduced to 24 h to reduce the confounding effect of different growth rates in control versus knockdown GBM cells, and comparisons were made between co-cultures with similar astrocyte:tumor cell ratios. Growth factor-reduced Matrigel (Corning) was used as a coating reagent to attach P3, BG5, BG7 and GG16 GBM cells and normal human astrocytes.

The following inhibitors were used: cytochalasin B (Sigma, C6762) and vincristine sulfate (Sigma, V8388). An equivalent amount of vehicle (DMSO) was used as a control. Cell viability was assessed with a LIVE/DEAD Fixable Blue Dead Cell staining kit.

For viability testing of P3 in the presence of inhibitor, GBM cells were seeded at 5,000 cells per Matrigel-coated well of a 96-well plate in 150 µl of complete Neurobasal medium. After incubation with cytochalasin B for 24 h, cell proliferation reagent WST-1 (Roche, CELLPRO-RO) was added, and sample absorbance measurements at 450 nM were determined on a multiscan FC microplate photometer (Thermo Scientific).

For transwell experiments, human-derived GBM cells were plated on Geltrex-coated tissue culture wells. Under ‘contact’ conditions, human mito-mCherry astrocytes were simultaneously added to the culture well. Under ‘transwell’ conditions, an equal number of astrocytes was plated in a transwell insert (5-μm pore size, Corning), which was submerged in the culture medium of the underlying culture well.

Co-culture experiments were analyzed on a BD LSR Fortessa or BD FACS Symphony S6 (BD Biosciences) operated by BD FACSDiva software (v8.0 or v9.0). FlowJo software (BD Biosciences, v10.7.2 or 10.8.1) was used to analyze flow cytometry data. The gating strategy is described in Supplementary Fig. 1.

### Mouse tumor cell maintenance and transduction

SB28 cells were a gift from H. Okada (University of California, San Francisco). GL261 cells were obtained from the Developmental Therapeutics Program, National Cancer Institute. All cell lines were treated with 1:100 MycoRemoval Agent (MP Biomedicals) after thawing and were routinely tested for *Mycoplasma* spp. (Lonza). Cells were maintained in RPMI 1640 (Media Preparation Core, Cleveland Clinic) supplemented with 10% FBS (Thermo Fisher Scientific) and 1 U ml^–1^ penicillin + 1 μg ml^–1^ streptomycin (Cleveland Clinic Media Preparation Core), that is, non-stem-promoting conditions. For the generation of GFP-expressing GL261 cells, parental GL261 cells were transduced with pReceiver-Lv207 (Genecopoeia) and were selected with 300 μg ml^–1^ hygromycin B (Invitrogen). GFP expression was confirmed by flow cytometry.

### Mice

Tg(CAG-mKate2)1Poche/J (mito::mKate2; stock 032188) mice were purchased from The Jackson Laboratory and were housed in the Cleveland Clinic Biological Research Unit. Both sexes of mito::mKate2 mice were intracranially injected at 4–8 weeks of age with 10,000–20,000 SB28 or 100,000 GL261-GFP cells in 5 μl of RPMI null medium into the left cerebral hemisphere 2 mm caudal to the coronal suture and 3 mm lateral to the sagittal suture at a 90° angle with the skull to a depth of 2.5 mm using a stereotaxis apparatus (Kopf). Male NSG mice were bred in-house by the Cleveland Clinic Biological Resources Unit, and tumors were established orthotopically as described above. Mice were fed standard chow (Teklad Global 18% Protein Rodent Diet, 2913, Envigo) and filtered water ad libitum and were housed in forced/filtered air isolator cages containing up to five mice. Mice were maintained on a 12-h light/12-h dark cycle, with a temperature of 20–26 °C and humidity of 30–70%.

### Bone marrow transplantation

Four-week-old male mice were treated with 11 Gy radiation in two fractions 3–4 h apart. Reconstitution was achieved by retro-orbital injection of 2 × 10^6^ bone marrow cells from mito::mKate2 mice. Drinking water was supplemented with Sulfatrim (trimethoprim–sulfamethoxazole; Pharmaceutical Associates) during the first 10 d, and mice were monitored for an additional 6 weeks for weight loss and symptoms of infection before tumor inoculation.

### Orthotopic xenograft models in nude rats

Immunodeficient nude-RNU rats of both sexes, bred in-house, were fed a diet containing standard pellets (Sniff, V1536-000), had access to water ad libitum and were housed in filtered air isolator cages (Allentown type IV (Rat 1800), HEPA filter) with a 12-h light/12-h dark cycle at 21 °C and ~45% humidity. Stereotactical implantation of tumor cells into the brain has been described previously^57^. To model mitochondria transfer from normal cells to tumor cells, a high-titer mitoDsRed lentivirus was injected into the brain followed by implantation of GFP^+^ or mito-GFP^+^ tumor acceptor cells after 7 d. Animals were euthanized with CO_2_ and perfused with 0.9% NaCl.

### Immunofluorescence

For immunofluorescence analysis, brains were fixed in 4% paraformaldehyde (PFA), dehydrated in a 30% sucrose solution, embedded in optimal cutting temperature compound and snap frozen. Sectioning (10 μm thick) was performed on a Leica CM3050 S precooled to −20 °C before use. Sections were protected from light and frozen at −80 °C. For staining, tissue sections were incubated overnight at 4 °C with the following primary antibodies: monoclonal anti-GFAP, anti-human nestin, anti-F-actin, anti-GAP43, chicken anti-GFP, rabbit anti-phospho-histone H3 and rabbit anti-cleaved caspase-3. The following secondary antibodies were used: goat anti-mouse 647 at room temperature for 120 min, donkey anti-chicken Alexa Fluor 488 overnight at 4 °C and WGA Alexa Fluor 680 (4 μg ml^–1^ in HBSS-T with magnesium and calcium) for 1 h at room temperature.

Cells on coverslips were fixed in 4% PFA and permeabilized with 0.1% Triton X-100 (Sigma) for 20 min at room temperature. Anti-TOMM20 or anti-nestin was incubated overnight at 4 °C. Goat anti-mouse 488 or goat anti-rabbit 647 were incubated for 60 min at room temperature.

### Confocal microscopy and time-lapse imaging

Confocal microscopy of stained mouse tissue sections was performed using a Leica SP8. Still-image processing and *z* reconstructions were completed using LasX software (version 3.3; Leica). Image analysis for estimation of mito::mKate2 transfer to GBM cells in vivo was performed using Velocity software (version 6.3; PerkinElmer). The three-dimensional (3D) segmentation algorithms (Supplementary Note) were set to minimize the (1) detection of mKate2 channel noise (using wild-type tissue sections as a negative control) and (2) identification of GFP^+^ cells not morphologically compatible with tumor cells, likely the result of GFP phagocytosis in the TME (using a relevant size cutoff).

SB28 cells (40,000) were cocultured with 80,000 mito::mKate2 astrocytes in a glass-bottom 35-mm dish (Mat-tek) overnight. Growth medium was replaced with phenol red-free NSC medium. Multiple full-thickness *z* stacks were obtained every 10 min using a Leica SP8 microscope in a 37 °C chamber supplemented with 5% CO_2_ and 95% humidity using a ×20/0.8-NA objective lens. Time-lapse frames were subsequently analyzed by LasX software.

L1 and DI318 cells were stained with CellTrace Green (Thermo Fisher) at 5 μM in serum-free medium at 37 °C for 30 min and washed with complete Neurobasal medium before plating with human mito-mCherry astrocytes in ibidiTread 35-mm microscopy dishes (Ibidi) that had been coated with Geltrex (Gibco). After 48 h, cells were washed gently with PBS, fixed with 4% PFA for 15 min at 4 °C and imaged by confocal microscopy.

For TOMM20 colocalization experiments, cells were plated as described above and cocultured for 72 h. Cells were then stained with primary antibody (anti-TOMM20; clone D8T4N) for 1 h at room temperature and with secondary anti-rabbit DyLight405 alpaca for 1 h at room temperature. Colocalization analysis (line profile quantification) was performed using Image-Pro Plus 10 (Media Cybernetics).

Confocal imaging for P3, BG5, BG7 and GG16 co-culture experiments was performed using a Leica TCS SP8 STED 3X (Leica Microsystems) run by LasX software (version 3.3, Leica, version LAS4.13). Image analyses were performed with ImageJ (v2.3.0/1.53f) and Imaris (v9.6).

### Measurement of mitochondrial trafficking in vivo by flow cytometry

Resected tumors or the contralateral hemisphere were digested with 1 mg ml^–1^ collagenase IV (StemCell Technologies) and 1 mg ml^–1^ DNase I (Roche) for 15 min at 37 °C. Samples were strained through a 100-μm strainer (Fisherbrand) and washed with PBS. Cells were stained with a LIVE/DEAD Fixable Blue Dead Cell Stain kit (Thermo Fisher Scientific) for 10 min on ice, treated with FcR blocking reagent (Miltenyi Biotec) diluted 1:50 for 15 min on ice and stained with 1:100 APC-conjugated anti-CD11b (BioLegend, clone M1/70) for 20 min to exclude phagocytic cells. Samples were fixed overnight with a eBioscience FoxP3 transcription factor fixation kit (Thermo Fisher Scientific) and analyzed with a BD LSRII Fortessa (BD Biosciences) in PBS.

### Generation of mouse astrocytes and microglia and in vitro mouse mitochondria transfer assay

Brain-resident glial cell cultures were obtained, as previously described^59^, by resecting the subventricular zone of brains from postnatal day 0–3 mice and culturing in NSC medium. Microglia were generated by culturing confluent monolayers of early passage (passage 2 or 3) glial cell cultures with microglia polarization medium (DMEM-F12, 1 U ml^–1^ penicillin + 1 μg ml^–1^ streptomycin, 10% FBS and 20 ng ml^–1^ granulocyte–macrophage colony-stimulated factor (BioLegend)). After 5 d, microglia (loosely adherent) were obtained by agitation of the culture flask on an orbital shaker for 45 min.

A total of 20,000–40,000 astrocytes and microglia were separately cultured in a 96-well flat-bottom plate in NSC medium or microglia polarization medium, respectively. Forty-eight hours later, supernatants were collected and centrifuged at 400*g* for 5 min to remove residual cells; these supernatants consisted of the conditioned, cell-free culture medium. Tumor cells were added at a recipient:donor ratio of 2:1 to the adherent donor cell cultures to assess total mitochondria transfer (contact dependent and independent); separately, tumor cells alone were cultured with conditioned, cell-free medium described above to assess contact-independent (secreted) mitochondria transfer. Samples were incubated for 2 h and treated with Accutase to generate single-cell suspensions. Cells were transferred to 96-well U-bottom plates to stain with LIVE/DEAD dye. Exogenous mitochondria uptake by GFP^+^ tumor cells was assessed with a BD LSRII Fortessa.

### Generation of mouse macrophages and in vitro mitochondria uptake assay

Bone marrow from the femurs and tibiae of 4- to 8-week-old male and female mito::mKate2 mice was flushed with PBS using a 27-gauge needle. Eighty thousand cells were cultured in 24-well plates and treated with 50 ng ml^–1^ recombinant mouse macrophage colony-stimulating factor (BioLegend) in IMDM (Media Preparation Core) supplemented with 1 U ml^–1^ penicillin + 1 μg ml^–1^ streptomycin and 20% FBS for 6 d. Interferon-γ or interleukin-4 (50 ng ml^–1^; BioLegend) was added for 48 h to further induce polarization of macrophages to M1- or M2-like macrophages, respectively. Supernatants were collected and centrifuged at 400*g* for 5 min to remove residual cells and to generate conditioned, cell-free culture medium. Tumor cells were added at twofold abundance in technical duplicates for a 2-h incubation, as described above, to test contact-dependent versus contact-independent mitochondria transfer. Samples were incubated with Accutase for 5 min and transferred into 96-well U-bottom plates for staining with the viability dye and anti-CD11b as described above. Exogenous mitochondria uptake was analyzed in GFP^+^ tumor cells using a BD LSRII Fortessa.

### Western blotting

Cells were washed twice with PBS and dissolved in lysis buffer (10 mM Tris-HCl (pH 7.4), 150 mM NaCl, 0.5% NP-40, 1% Triton X-100 and 1 mM EDTA) supplemented with protease and phosphatase inhibitor cocktails (Roche). Protein concentration was quantified by Bradford assay (Euromedex). Cell lysates were resuspended in Laemmli buffer (62.5 mM Tris (pH 6.8), 10% glycerol, 2.5% SDS and 2.5% β-mercaptoethanol). The primary antibody was anti-GAP43, the housekeeping protein vinculin was used as the loading control and the secondary antibody was goat anti-rabbit horseradish peroxidase (HRP; Invitrogen, 31462). Membranes were developed on a LAS 3000 (version 2.2; Fujifilm).

For GAP43 blots (Extended Data Fig. 6i,j) and stem cell transcription factors (Extended Data Fig. 10b), cells were lysed with ice-cold RIPA buffer. The following primary antibodies were used: anti-GAP43, anti-actin, anti-SOX2 and anti-OCT4. Secondary antibodies were goat anti-rabbit HRP or goat anti-mouse HRP.

### Sorting of tumor cells from co-cultures

For mouse GBM models, astrocytes were collected from flasks by Accutase treatment and stained with a 1:1,000 dilution of CellTrace Violet cell proliferation dye in PBS at 37 °C for 20 min. Tumor cells and astrocytes were then cocultured at a 1:1 ratio for 48 h in NSC medium. Samples were sorted into RPMI with 20% FBS and cultured overnight in complete RPMI for subsequent functional assays.

For collection of double-positive or single-positive cells in co-cultures of P3, BG5 and GG16 GBM cells with immortalized mitoDsRed normal human astrocytes by cell sorting, 2 × 10^6^ cells of each cell line were seeded in Neurobasal medium on Matrigel (T75 flasks). For analyzing mitochondria transfer by flow cytometry, 1.5 × 10^5^ cells from each cell line were seeded in Neurobasal medium on Matrigel (T25 flasks). L1 and DI318 cells were sorted after 4 d of co-culture with immortalized mito-mCherry human astrocytes from Geltrex-coated flasks (seeded at 1:1 and 1:1.5 donor:recipient ratio, respectively, to adjust for differences in cell growth rate).

### In vitro limiting dilution assay

Sorted tumor cells were cultured at decreasing cell densities over 12 technical replicates in complete Neurobasal medium, and the number of wells containing spheres was counted after 11–14 d. For the mouse cell line SB28, 400 to 25 cells per well of a 96-well plate were seeded; for human lines, this ranged from 100 to 1.25 cells per well. The online ELDA tool (http://bioinf.wehi.edu.au/software/elda/, 24 October 2014 version) was used to calculate stem cell frequency^60^.

### In vivo limiting dilution/tumor initiation assays

Sorted mouse/human GBM cells with and without the acquisition of astrocyte mitochondria in vitro were counted with trypan blue using a TC-20 cell counter (Bio-Rad) and were volume adjusted to achieve decreasing cell concentrations (18,000–1,000, as indicated) for intracranial implantation. Subsequently, C57BL/6 mice (mouse GBM models) or NSG mice (human GBM models) were intracranially implanted with equal numbers of tumor cells, as described above. The identity of the implanted cells was then blinded to investigators. Mice were euthanized at humane endpoints (neurological symptoms, weight loss, poor grooming or any other sign of distress).

### Seahorse assay

For sorted P3 GFP mitoDsRed^+^ and mitoDsRed^−^ cells, mitochondrial respiration assays were performed using 3.0 mM oligomycin, 1.5 mM carbonyl cyanide m-chlorophenyl hydrazone (CCCP), 1.0 mM rotenone and 1.0 mM antimycin A in assay medium consisting of unbuffered, phenol red-free DMEM with 10 mM glucose, 2 mM sodium pyruvate and 4 mM l-glutamine with a pH of 7.4. Cells were incubated for 60 min in a Prep Station (Agilent) under non-CO_2_ conditions at 37 °C and were subsequently placed in the Seahorse Xfe96 analyzer. The chemical compounds were serially injected to manipulate the cells and create metabolic flux reports. Data were analyzed using Agilent Seahorse Wave Controler v2.6.3, IDEAS software (version 6.2; EMD Millipore).

### ATP quantification

CellTiter-Glo reagent (100 µl; Promega) was added to cells and incubated on an orbital shaker at room temperature for 20–30 min. ATP levels were quantified indirectly by relative luminescence intensity measured on a Victor 3 plate reader (PerkinElmer).

### Metabolic protein flow cytometry analysis

For mouse cells, the metabolic protein flow cytometry panel was adapted from Ahl et al.^40^. CellTrace Violet-stained astrocytes were cocultured with tumor cells overnight at a 1:1 ratio. Samples were fixed in eBioscience FoxP3 transcription factor fixation buffer for 30 min on ice and stained with the following antibodies in 1× permeabilization buffer for 30 min at room temperature: anti-argininosuccinate synthetase 1 (ASS1), anti-ATP synthase F1 subunit α (ATP5A), anti-glucose transporter 1 (GLUT1), anti-isocitrate dehydrogenase 2 (IDH2), anti-glucose 6 phosphate dehydrogenase (G6PD), anti-acetyl-CoA carboxylase (ACC1), anti-peroxiredoxin 2 (PRDX2), anti-hexokinase 1 (HK1), anti-carnitine palmitoyltransferase I (CPT1A), anti-SLC20A1, anti-mouse IgG1 (κ monoclonal), anti-mouse IgG2b (κ monoclonal) and rabbit IgG (monoclonal). Samples were washed and resuspended in 1× permeabilization buffer containing goat anti-mouse IgG H&L (Alexa Fluor 647) or donkey anti-rabbit IgG H&L (Alexa Fluor 647). After 30 min of incubation at room temperature, cells were washed with permeabilization buffer and resuspended in PBS for analysis with a BD LSRII Fortessa. Geometric mean fluorescence intensity was used to calculate expression levels after subtraction of the background levels from isotype control staining.

The same procedure was used for human cell metabolic flow, with minor modifications. Seven different human-derived cell lines were cocultured with human mito-mCherry astrocytes for 4 d. Most antibodies listed above cross-reacted with human antigens and were thus also used in these experiments, with the exception of using directly conjugated Alexa Fluor 647 antibodies for ATP5A and GLUT1.

### Metabolomics analysis

Sorted mouse GBM cell pellets were resuspended in 80% ice-cold methanol for metabolite extraction and sent to an academic core facility for targeted metabolomics (Beth Israel Deaconess Mass Spectrometry Core Facility, Harvard Medical School). Data were normalized by peak count. Values of zero (below the limit of quantitation) were substituted with the approximate limit of quantitation (2,000 peak count) to facilitate fold change analysis. Metabolites enriched by >20% in mKate2^+^ GBM cells were put into the metabolic pathway enrichment analysis algorithm MetaboAnalyst 5.0 web tool (www.metaboanalyst.ca)^61^. The following analysis settings were used: HMDB and KEGG compound names; feature type = metabolites; KEGG analysis.

For human GSCs, triplicate cell pellet samples were lysed in 80:20 methanol:water at dry ice temperature. The quantity of the metabolite fraction analyzed was adjusted to the cell count. Extracts were clarified by centrifugation, dried by nitrogen blower and reconstituted in equal volumes 50:50 methanol:water. Metabolite fractions were analyzed by targeted liquid chromatography–tandem mass spectrometry via dynamic multiple reaction monitoring. An Agilent Technologies Triple Quad 6470 liquid chromatography–tandem mass spectrometry system, consisting of the 1290 Infinity II LC flexible pump (Quaternary Pump), the 1290 Infinity II multisampler, the 1290 Infinity II multicolumn thermostat with six-port valve and the 6470 triple quad mass spectrometer, was used for analysis. Agilent MassHunter Workstation software LC/MS data acquisition for 6400 Series Triple Quadrupole MS with version B.08.02 was used for compound optimization and sample data acquisition. Studies were performed in negative ion acquisition mode with ion-pairing chromatography using an Agilent ZORBAX RRHD Extend-C18, 2.1 × 150 mm, 1.8 µm and ZORBAX Extend Fast Guards for UHPLC separation. Agilent MassHunter Workstation quantitative analysis for QQQ version 10.1, build 10.1.733.0, was used to integrate and quantitate metabolite peak areas. Liquid chromatography–mass spectrometry peaks corresponding to metabolites with coefficients of variation greater than 0.5 underwent manual inspection and integration. The data were normalized to the average sum of metabolites from all the samples and were analyzed using Morpheus to generate the heat map. Metaboanalyst was used to compare metabolites and metabolic pathways enriched in mito-mCherry^+^ L1 cells and mito-mCherry^–^ L1 cells.

### Phosphoprotein array

Human-derived GBM cells (L1) were cocultured with human mito-mCherry astrocytes for 4 d and flow sorted to obtain mCherry^+^ and mCherry^–^ GBM cells. Sorted cell pellets were flash-frozen in liquid nitrogen and sent for a commercial microarray platform, antibody-based phosphoprotein array (Phospho Explorer array, FullMoon BioSystems). The complete antibody list can be found in Supplementary Table 3. Analysis was conducted using the phosphorylation ratio of each protein for samples tested (phosphorylated protein signal divided by corresponding total protein signal). We analyzed the functional enrichment of the upregulated phosphoproteins using Enrichr^62^ against the Gene Ontology (GO) biological process term data set. GO terms with FDR values of <0.05 were considered significantly enriched. We summarized the enriched terms using Revigo^63^ to generate a graph-based view of the subdivisions of the terms.

### ImageStream

A clone of HEK293T cells able to grow in serum-free medium (CSC293T) was generated and cultured as previously described^64^. CSC293T cells were transfected with psPAX2, pCMV-VSVG and pLYS1-Mito-GFP or pCMV-RFP using FuGENE HD transfection reagent (Promega). Viral concentration was determined using the Lenti-X qRT–PCR titration kit (Takara Bio). Immortalized normal human astrocytes were transduced with mito-GFP lentivirus and selected for cells stably expressing mito-GFP using puromycin. D456 and JX22 cells were transduced with RFP lentivirus and selected using blasticidin S (Gibco) to select for stable RFP-expressing cells. Where indicated, cells were sorted for mito-GFP positivity or RFP positivity with assistance from the Flow Cytometry Core at the University of Alabama at Birmingham.

RFP-expressing D456 or JX22 cells were cocultured at a 1:1 ratio in the presence of mito-GFP^+^ astrocytes for 24 h. Samples were imaged at ×40 magnification with extended depth of field. Mito-GFP was acquired on ch02, and RFP was acquired on ch04. Ch01 and ch09 were used for brightfield imaging, and ch12 was used for side scatter. Five thousand events were recorded, and relevant single-color and unstained controls were used. Data were analyzed using IDEAS software (version 6.2; EMD Millipore).

### RNA-seq

mKate^+^ and mKate^–^ SB28 cells and astrocytes from three distinct co-cultures (biological replicates) were sorted into multiple 1.5-ml DNA LoBind microtubes (Eppendorf), each containing 700 μl of RLT Plus lysis buffer (Qiagen) supplemented with 1% β-mercaptoethanol. RNA isolation was performed using the RNEasy Plus Micro kit (Qiagen).

RNA-seq and analysis were performed by GENEWIZ. Briefly, samples were sequenced using an Illumina HiSeq, with a 2 × 150 base pair configuration and ≥350 million raw paired-end reads. An average of 41.6 million paired-end reads was sequenced across nine samples. After Illumina universal adapters were trimmed, the reads were mapped to the *Mus musculus* GRCm38 reference genome using the STAR aligner v.2.5.2b. Unique gene hit counts were calculated by using featureCounts from the Subread package v.1.5.2.

For comparison of tumor cells with astrocytes, genes with an adjusted *P* value of <0.05 and absolute log_2_ (fold change) of >1 were called as differentially expressed genes (DEGs) using DESeq2. For assessment of differentially upregulated pathways in mKate^+^ versus mKate^–^ SB28 cells, genes that were upregulated >1.5-fold with a count number >50 and an unadjusted *P* value of <0.05 (Supplementary Table 1) were plugged into https://maayanlab.cloud/Enrichr/.

### Protein–protein interactions and network visualization

Differential expression analysis was performed using edgeR 3.34 (ref. ^65^). Genes with a count per million greater than 1 in at least two samples were used for the analysis. *P* values of <0.05 were considered significant. The mouse DEGs were mapped to the human homologs using the NCBI HomoloGene database (https://www.ncbi.nlm.nih.gov/homologene). We then performed the enrichment analysis using Enrichr^62^ for the entire set of DEGs and for the up- and downregulated genes separately.

The protein–protein interactions among the DEGs were extracted using a human protein interactome we built previously^66^ that contains 17,706 protein nodes and 351,444 protein–protein interaction edges. We then visualized this protein–protein interaction network using Cytoscape 3.8 (ref. ^67^). Genes that localize to mitochondria are indicated by a diamond node shape based on the Human MitoCarta2.0 database^68^.

### Cell cycle analysis

Single-cell suspensions (from mouse tumors or cultured cells) were fixed in PBS with 4% PFA for 1 h on ice and washed with permeabilization buffer (Foxp3/transcription factor staining buffer set; Ebioscience). Subsequently, cells were stained with Hoechst 33342 (Thermo Fisher) at 3.33 μg ml^–1^ in PBS for 1 h at room temperature, washed with PBS supplemented with 2% bovine serum albumin and assayed by flow cytometry.

### Mitochondria isolation

Mitochondria were isolated from confluent immortalized human astrocyte cultures expressing mCherry by using the mitochondria isolation kit for cultured cells (Thermo Fisher Scientific), as per the manufacturer’s instructions. Mitochondria were quantified by total protein using the Qubit protein assay kit (Thermo Fisher), according to the manufacturer’s protocol.

### Quantification of phospho-histone H3 and cleaved caspase-3 immunofluorescence

To quantify elements within the TME, tissue was examined from three animals per biological sex per mitochondrial transfer status. For a given animal, 15 representative images were captured (5 images × 3 tumor-bearing sections). Cellular proliferation was quantified by counting each phosphorylated histone H3^+^ nucleus in each visual field. This count was divided by the fluorescence intensity of the GFP signal of the same field to account for variation in tumor size and cellular density to yield a mitotic index. The apoptotic cell death index was quantified by dividing the fluorescence intensity of the cleaved caspase-3 signal by the fluorescence intensity of the GFP signal in the same visual field.

### MT quantifications

MT number was quantified manually using the NIH ImageJ software. DAPI and nestin-immunostained confocal images magnified ×200 were used. For each experimental condition, a minimum of 80–120 MTs showing a direct connection between two tumor cells was included for measurement. All measured data were exported into Microsoft Excel and GraphPad Prism 8.1.2 for further calculation of statistical significance.

### Statistics and reproducibility

For most in vitro experiments, a minimum of three biologically independent samples was used per experimental group; power was not calculated. For in vivo experiments not involving tumor initiation capacity, sample size was determined based on minimum utilization of vertebrate animals and high expected magnitude of effect without formal power evaluation. For in vivo experiments involving tumor initiation analysis, sample size was determined by prior experience and application of this assay in stem cell biology studies, for example, in Karunanithi et al.^69^. No data were excluded from analyses. Key findings were replicated across time, by different institutions, using diverse models. For survival/tumor initiation studies, mice were randomized before tumor implantation, and investigators were blinded to the grouping. Data distribution was assumed to be normal, but this was not formally tested.

### Data representation and analysis

Flow cytometry data were analyzed and generated using FlowJo software (BD Biosciences, v10.7.2). Graphs were generated and statistical analyses were performed using Excel (Microsoft Office, v16.52) or Prism (GraphPad, v9.2.0) software. All measurements shown represent distinct samples, unless otherwise indicated. All statistical tests are two tailed and corrected for multiple comparisons, unless otherwise indicated.

### Reporting summary

Further information on research design is available in the Nature Portfolio Reporting Summary linked to this article.

## Supplementary information

Supplementary Information Supplementary Note and Fig. 1.

Reporting Summary

Supplementary Tables 1–4 Supplementary Tables 1–4 (in spreadsheet tabs) with additional information for RNA-seq, metabolomics, phosphoprotein array and shRNA constructs.

Supplementary Video 1 Human-derived GSCs (L1) internalize mito-mCherry astrocyte-derived mitochondria; cyan, TOMM20 (mitochondrial marker); magenta, mito-mCherry. Cyan cells comprise GSCs. Magenta cells comprise mito-mCherry astrocytes. Mito-mCherry punctae can be seen within the GSCs, denoting astrocytic mitochondria internalized in GSCs. See also Extended Data Fig. 5a for still images with accompanying *z* reconstructions.

Supplementary Video 2 Human-derived GSCs (DI318) internalize mito-mCherry astrocyte-derived mitochondria; cyan, TOMM20 (mitochondrial marker); magenta, mito-mCherry. Cyan cells comprise GSCs. Magenta cells comprise mito-mCherry astrocytes. Mito-mCherry punctae can be seen within the GSCs, denoting astrocytic mitochondria internalized in GSCs. See also Extended Data Fig. 5a for still images with accompanying *z* reconstructions.

Supplementary Video 3 Time-lapse confocal images of in vitro mitochondria transfer. Astrocytes from mito::mKate2 mice were cocultured with GFP-expressing SB28 cells for 16 h before live imaging. Full-thickness *z* stacks were obtained at 10-min intervals. The movie depicts a mito::mKate2^+^ astrocyte (magenta) in contact with a GFP-expressing SB28 cell (green). An mKate2^+^ mitochondrion is transferred to the dividing GBM cell, which shuttles the mitochondrion back and forth along an intercellular connection linking the GBM daughter cells. At the end of the video, the mKate2^+^ mitochondrion is retained by the lower daughter cell. See also Extended Data Fig. 6c for still images with accompanying *z* reconstructions.

## Data Availability

Sequencing files have been deposited to Gene Expression Omnibus under accession number GSE183004. Metabolic pathway analysis was based on the KEGG human metabolic pathways database (October 2019; https://www.genome.jp/kegg/pathway.html#metabolism). Protein phosphophorylation array data were mapped to pathways based on the GO biological process term data set (http://geneontology.org/). RNA-seq reads of mouse cells were mapped to the *M. musculus* GRCm38 reference genome (https://www.ncbi.nlm.nih.gov/assembly/GCF_000001635.20/). The RNA-seq inferred protein–protein interaction network was constructed by mapping to human homologs using the NCBI HomoloGene database (https://www.ncbi.nlm.nih.gov/homologene). Genes encoding mitochondria-localizing proteins were identified with MitoCarta2.0 (https://www.broadinstitute.org/files/shared/metabolism/mitocarta/human.mitocarta2.0.html). All other data are available in the main text or the Supplementary Information. Source data are provided with this paper.

## Source data

Source Data Fig. 1 Statistical source data.
Source Data Fig. 2 Statistical source data.
Source Data Fig. 3 Statistical source data.
Source Data Fig. 4 Statistical source data.
Source Data Fig. 5 Statistical source data.
Source Data Fig. 6 Statistical source data.
Source Data Fig. 7 Statistical source data.
Source Data Extended Data Fig. 3 Statistical source data.
Source Data Extended Data Fig. 4 Statistical source data.
Source Data Extended Data Fig. 5 Statistical source data.
Source Data Extended Data Fig. 6 Statistical source data.
Source Data Extended Data Fig. 6 Unprocessed western blots.
Source Data Extended Data Fig. 7 Statistical source data.
Source Data Extended Data Fig. 8 Statistical source data.
Source Data Extended Data Fig. 9 Statistical source data.
Source Data Extended Data Fig. 10 Statistical source data.
Source Data Extended Data Fig. 10 Unprocessed western blots.
