# Supplementary material for: GAP43-dependent mitochondria transfer from astrocytes enhances glioblastoma tumorigenicity
Source: Nat Cancer. 2023 May 11;4(5):648–64. doi: 10.1038/s43018-023-00556-5 (PMC10212766; doi:10.1038/s43018-023-00556-5)
Supplement: Supplementary file 1 — Supplementary Note and Fig. 1. [file 43018_2023_556_MOESM1_ESM.pdf]

nature cancer

Article

https://doi.org/10.1038/s43018-023-00556-5

GAP43-dependent mitochondria transfer from astrocytes enhances glioblastoma tumorigenicity

In the format provided by the authors and unedited

Table of Contents

Supplementary Note 3
Supplementary Fig. 1. Gating strategy 4

Supplementary Note

GL261 model

(1) Define GL261 objects:
- a. Channel 3 (GFP) threshold
  - i. intensities: 11-185
  - ii. Minimum object size = 10000 cubic microns
- b. Fill holes in objects
- c. Separate touching objects
  - i. Object size guide = 1200 cubic microns

(2) Define mitochondria objects
- a. Channel 1 (mito::mKate2) threshold
  - i. Intensities 20-255
  - ii. Minimum object size = 1 cubic micron
- b. Separate touching objects
  - i. Object size guide = 25 cubic microns
- c. Filter population
  - i. Volume >= 0.1 cubic microns

(3) Compartmentalize mitochondria objects inside GL261 objects

SB28 model

(1) Define SB28 objects:
- a. Channel 3 (GFP) threshold
  - i. intensities: 20-255
  - ii. Minimum object size = 10000 cubic microns
- b. Fill holes in objects
- c. Separate touching objects
  - i. Object size guide = 1200 cubic microns
- d. Filter population
  - i. Volume > 600 cubic microns

(2) Define mitochondria objects
- a. Channel 1 (mito::mKate2) threshold
  - i. Intensities 20-255
  - ii. Minimum object size = 1 cubic micron
- b. Separate touching objects
  - i. Object size guide = 25 cubic microns
- c. Filter population
  - i. Volume >= 0.1 cubic microns

(3) Compartmentalize mitochondria objects inside SB28 objects

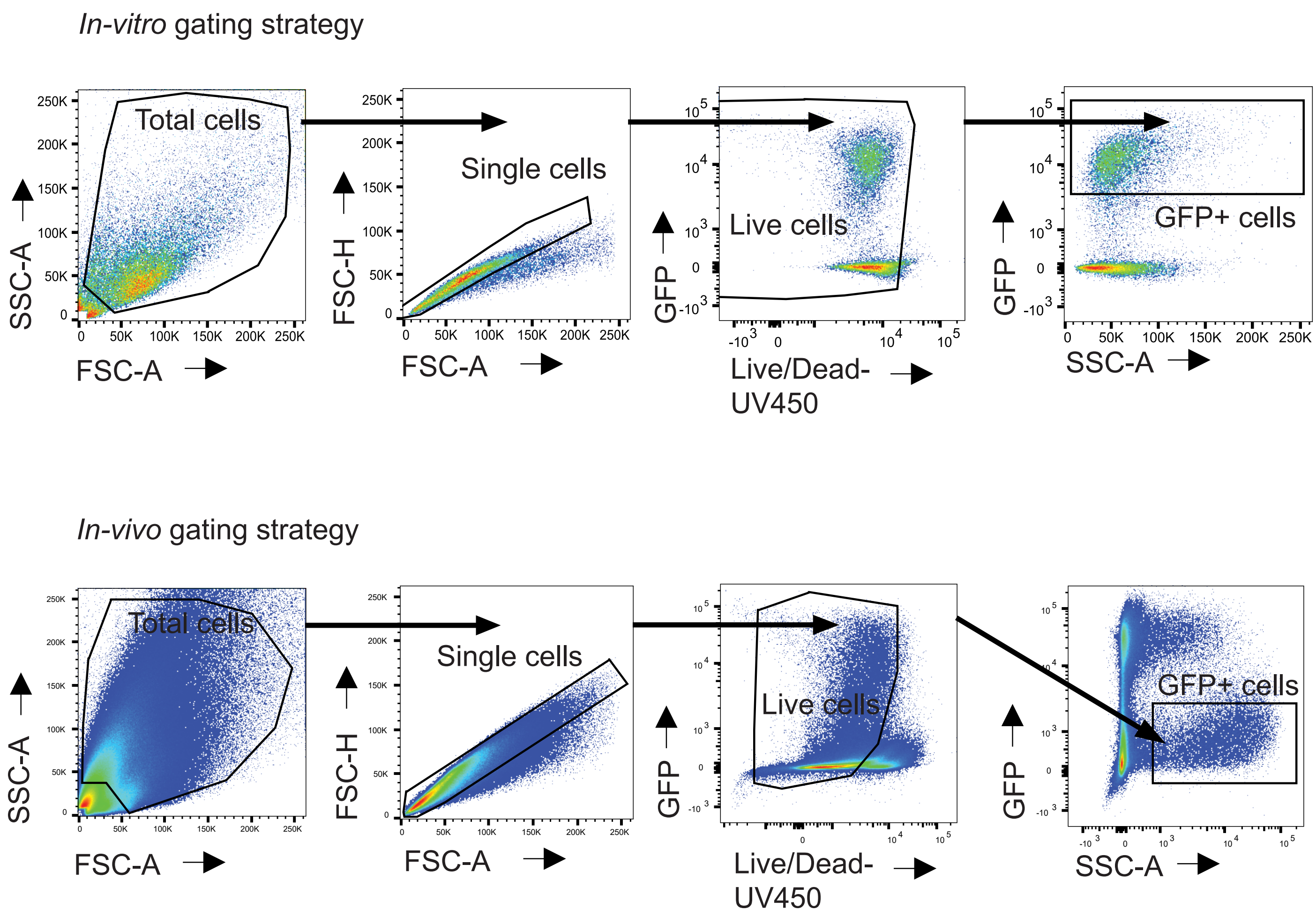

Supplementary Fig. 1. Gating strategy

Flow cytometry gating strategy used for *in vitro* and *in vivo* experiments.
