# Supplementary material for: GAP43-dependent mitochondria transfer from astrocytes enhances glioblastoma tumorigenicity
Source: Nat Cancer. 2023 May 11;4(5):648–64. doi: 10.1038/s43018-023-00556-5 (PMC10212766; doi:10.1038/s43018-023-00556-5)
Supplement: Supplementary file 2 — Reporting Summary [file 43018_2023_556_MOESM2_ESM.pdf]

Corresponding author(s): Justin D. Lathia and Hrvoje Miletic

Last updated by author(s): Mar 2, 2023

Reporting Summary

Nature Portfolio wishes to improve the reproducibility of the work that we publish. This form provides structure for consistency and transparency in reporting. For further information on Nature Portfolio policies, see our Editorial Policies and the Editorial Policy Checklist.

Statistics

For all statistical analyses, confirm that the following items are present in the figure legend, table legend, main text, or Methods section.

n/a	Confirmed	
☐	☒	The exact sample size (*n*) for each experimental group/condition, given as a discrete number and unit of measurement
☐	☒	A statement on whether measurements were taken from distinct samples or whether the same sample was measured repeatedly
☐	☒	The statistical test(s) used AND whether they are one- or two-sided<br>*Only common tests should be described solely by name; describe more complex techniques in the Methods section.*
☒	☐	A description of all covariates tested
☐	☒	A description of any assumptions or corrections, such as tests of normality and adjustment for multiple comparisons
☐	☒	A full description of the statistical parameters including central tendency (e.g. means) or other basic estimates (e.g. regression coefficient) AND variation (e.g. standard deviation) or associated estimates of uncertainty (e.g. confidence intervals)
☐	☒	For null hypothesis testing, the test statistic (e.g. *F*, *t*, *r*) with confidence intervals, effect sizes, degrees of freedom and *P* value noted<br>*Give P values as exact values whenever suitable.*
☒	☐	For Bayesian analysis, information on the choice of priors and Markov chain Monte Carlo settings
☒	☐	For hierarchical and complex designs, identification of the appropriate level for tests and full reporting of outcomes
☒	☐	Estimates of effect sizes (e.g. Cohen's *d*, Pearson's *r*), indicating how they were calculated

Our web collection on statistics for biologists contains articles on many of the points above.

Software and code

Policy information about availability of computer code

Data collection	BD LSR Fortessa/ BD FACS Symphony S6, FACS Diva v8.0 and v9.0 ; Agilent Seahorse Wave Controler v2.6.3, IDEAS software (version 6.2; EMD Millipore), LasX software (version 3.3, Leica and version LAS4.13), Fusion (Version 2020.2012.7), WorkOut (Version 2.0), LAS-3000 –(version 2.2)
Data analysis	FlowJo software (BD Biosciences, v10.7.2 or 10.8.1) was used to analyze flow cytometry data. ImageStream data was analyzed using IDEAS software (version 6.2; EMD Millipore). Image analyses were also performed with ImageJ (v2.3.0/1.53f) and Imaris (v9.6). Graphs were generated and statistical analysis were performed using Excel (Microsoft Office, v16.52), Prism (GraphPad, v9.1.0 or v9.2.0) or RStudio (v2022.02.1) software. ELDA tool (http://bioinf.wehi.edu.au/software/elda/) . LasX software (version 3.3, Leica). Velocity software (version 6.3, PerkinElmer)<br><br>After Illumina sequencing of universal adapters were trimmed, the reads were mapped to the Mus musculus GRCm38 reference genome using the STAR aligner v.2.5.2b. Unique gene hit counts were calculated by using featureCounts from the Subread package v.1.5.2. Differential expression analysis was performed using edgeR 3.34. The mouse differentially expressed genes (DEGs) were mapped to the human homologs using the NCBI HomoloGene database (https://www.ncbi.nlm.nih.gov/homologene). We then performed the enrichment analysis using Enrichr for the entire DEGs and for the up- and down-regulated genes separately. The protein-protein interactions (PPIs) among the DEGs were extracted using a human protein interactome we built previously (citation in main text) that contains 17,706 protein nodes and 351,444 PPI edges. We then visualized this protein-protein interaction network using Cytoscape 3.8. Genes that localize to mitochondria are indicated by diamond node shape based on the Human MitoCarta2.0 database.<br><br>Mouse metabolomics: Data were normalized by peak count. Values of zero (below limit of quantitation) were substituted with the approximate limit of quantitation (2000 peak count) to facilitate fold-change analysis. Metabolites enriched by >20% in mKate2+ GBM cells

were put into the metabolic pathway enrichment analysis algorithm MetaboAnalyst 5.0 web tool (www.metaboanalyst.ca) . Settings for analysis were as follows: HMDB and KEGG compound names; feature type = metabolites; KEGG analysis.

Human metabolomics: Agilent MassHunter Workstation Quantitative Analysis for QQQ Version 10.1, Build 10.1.733.0 was used to integrate and quantitate metabolite peak areas. LC-MS peaks corresponding to metabolites with coefficients of variation greater than 0.5 underwent manual inspection and integration. The data was normalized to the average sum of metabolites from all the samples and analyzed using Morpheus to generate the heatmap, Metaboanalyst to compare metabolites and metabolic pathways enriched in mito-mCherry+ L1 cells and mito-mCherry- L1 cells.

Phospho-array: Analysis was conducted using the phosphorylation ratio of each protein for samples tested (phosphorylated protein signal divided by corresponding total protein signal). We analyzed the functional enrichment of the upregulated phosphoproteins using Enrichr against the Gene Ontology (GO) biological process term data set. GO terms with FDR < 0.05 were considered significantly enriched. We summarized the enriched terms using Revigo to generate a graph-based view of the subdivisions of the terms.

Citations for all analysis software algorithms used are provided in the manuscript.

Summary list of all software used:
FACS Diva v8.0 and v9.0
Agilent Seahorse Wave Controler v2.6.3
IDEAS software (version 6.2; EMD Millipore)
LasX software (version 3.3, Leica and version LAS4.13),
LAS-3000 –(version 2.2, Fujifilm)
Velocity software (version 6.3, PerkinElmer)
FlowJo software (BD Biosciences, v10.7.2 or 10.8.1)
ImagePro Plus 10 (Media Cybernetics)
ImageJ (v2.3.0/1.53f)
Imaris v9.6 (Oxford Instruments)
ELDA (October 24, 2014 version)
Agilent MassHunter Workstation Software LC/MS Data Acquisition for 6400 Series Triple Quadrupole MS with Version B.08.02
Agilent MassHunter Workstation Quantitative Analysis for QQQ Version 10.1, Build 10.1.733.0
Morpheus (Broad Institute; https://software.broadinstitute.org/morpheus/)
Revigo (http://revigo.irb.hr/)
STAR aligner v.2.5.2b
Subread package v.1.5.2
DESeq2 (no version number)
Enrichr (no version number)
edgeR 3.34
Cytoscape 3.8
Graphpad Prism 8.2.1 and 9.2.0
Microsoft Excel v16.52

For manuscripts utilizing custom algorithms or software that are central to the research but not yet described in published literature, software must be made available to editors and reviewers. We strongly encourage code deposition in a community repository (e.g. GitHub). See the Nature Portfolio guidelines for submitting code & software for further information.

Data

Policy information about availability of data

All manuscripts must include a data availability statement. This statement should provide the following information, where applicable:

- Accession codes, unique identifiers, or web links for publicly available datasets
- A description of any restrictions on data availability
- For clinical datasets or third party data, please ensure that the statement adheres to our policy

Sequencing files have been deposited to GEO with the accession number GSE183004. Metabolic pathway analysis was based on the KEGG human metabolic pathways database (Oct. 2019) (https://www.genome.jp/kegg/pathway.html#metabolism). Protein phospho array data was mapped to pathways based on the Gene Ontology (GO) biological process term data set (http://geneontology.org/). RNAseq reads of mouse cells were mapped to the Mus musculus GRCm38 reference genome (https://www.ncbi.nlm.nih.gov/assembly/GCF_000001635.20/). RNAseq inferred protein-protein interaction network was constructed by mapping to human homologs using the NCBI HomoloGene database (https://www.ncbi.nlm.nih.gov/homologene). Genes encoding mitochondria localizing proteins were identified with Mitocarta 2.0 (https://www.broadinstitute.org/files/shared/metabolism/mitocarta/human.mitocarta2.0.html). All other data are available in the manuscript and supplementary materials.

Human research participants

Policy information about studies involving human research participants and Sex and Gender in Research.

Reporting on sex and gender	Not applicable. De-identified patient-derived cell models were used.
Population characteristics	Not applicable
Recruitment	Not applicable
Ethics oversight	Patient material was obtained from surgeries performed at the Haukeland University Hospital (Bergen, Norway). Written

Ethics oversight	consent was obtained from patients with procedures that were approved for the projects (project numbers 013.09 and 151825) by the Regional Ethical Committee (Bergen, Norway). Animal experiments were approved by the Institutional Animal Care and Use Committee of Cleveland Clinic and local ethical committee (Bergen, Norway). Animals were treated in accordance with the Norwegian Animal Act.

Note that full information on the approval of the study protocol must also be provided in the manuscript.

Field-specific reporting

Please select the one below that is the best fit for your research. If you are not sure, read the appropriate sections before making your selection.

☒ Life sciences ☐ Behavioural & social sciences ☐ Ecological, evolutionary & environmental sciences

For a reference copy of the document with all sections, see nature.com/documents/nr-reporting-summary-flat.pdf

Life sciences study design

All studies must disclose on these points even when the disclosure is negative.

Sample size	For most in vitro experiments, a minimum of 3 independent samples was used per experimental group. This gave sufficient power to detect differences among groups with large magnitude of effects. The minimum sample size for RNAseq was limited to 3 due to practical considerations associated with labor, time and cost of sample generation and analysis. As indicated in the manuscript, this allowed only for hypothesis generation using differentially expressed genes detected by statistical comparisons without multiple comparison correction. For metabolic flow analysis, sample size was increased to 5-6 per group, as the expected difference in metabolic protein expression was smaller. For phospho-array, 3 independent biological samples were analyzed via pooled assay. for metabolomics analysis, 3 biological replicates were analyzed independently. For in vivo experiments, sample size was determined based on minimum utilization of vertebrate animals and high expected magnitude of effect.
Data exclusions	no data were excluded from analyses
Replication	Experiments were conducted across time, with primary cells derived from multiple animals, different cell passages/vials (cell lines), by >1 researcher. Findings were replicated across multiple models of GBM, and accross species (mouse, rat, human). Key findings were replicated across institutions by independent research teams (primarily Cleveland Clinic and University of Bergen). Metabolomics were conducted in two separate facilities for each of the mouse and human assays. In vivo experiments were replicated with multiple models (mouse, human). Findings were reproducible under these conditions.
Randomization	Mice were randomized prior to tumor implantation. Rats were not randomized as only one experimental group was used to show mitochondrial transfer in vivo. For in vitro data, randomization is not relevant as specific cell lines were used.
Blinding	For all survival studies, researchers were blinded to the group assignment of mice with intracranial tumors. In vitro experiments were performed by one person/experiment, so blinding was not possible.

Reporting for specific materials, systems and methods

We require information from authors about some types of materials, experimental systems and methods used in many studies. Here, indicate whether each material, system or method listed is relevant to your study. If you are not sure if a list item applies to your research, read the appropriate section before selecting a response.

Materials & experimental systems

n/a	Involved in the study	
☐	☒	Antibodies
☐	☒	Eukaryotic cell lines
☒	☐	Palaeontology and archaeology
☐	☒	Animals and other organisms
☒	☐	Clinical data
☒	☐	Dual use research of concern

Methods

n/a	Involved in the study	
☒	☐	ChIP-seq
☐	☒	Flow cytometry
☒	☐	MRI-based neuroimaging

Antibodies

Antibodies used	Antibody / Supplier / Clone / Cat. No / Lot no. / Dilution factor used<br>1 . chicken anti-GFP antibody / AvesLabs / AB_2307313 / GFP-1020 / GFP3717982 / 1000<br>2 . donkey anti-chicken AF488 / Jackson ImmunoResearch / AB_2340375 / 703-545-155 / 162189 / 500<br>3 . APC-conjugated anti-CD11b antibody / Biolegend / M1/70 / 101212 / B288782 / 100<br>4 . ASS1 / Abcam / 2B10 / ab124465 / GR3209494-9 / 200<br>5 . anti-ATP synthase F1 subunit alpha (ATP5A) / Abcam / 7H10BD4F9 / ab110273 / GR3242541-11 / 1000

6 . anti-glucose transporter 1 (GLUT1) / Abcam / EPR3915 / ab115730 / GR3266142-11 / 40
7 . anti-glucose 6 phosphate dehydrogenase (G6PD) / Abcam / EPR20668 / ab210702 / GR3218248 / 400
8 . anti-Acetyl-CoA carboxylase (ACC1) / Abcam / EPR23235-147 / ab269273 / GR3340965-3 / 1000
9 . anti-peroxiredoxin 2 (PRDX2) / Abcam / EPR5154 / ab109367 / GR3257260-4 / 200
10 . anti-hexokinase 1 (HK1) / Abcam / EPR10134(B) / ab150423 / GR105890-12 / 20
11 . anti-Carnitine palmitoyltransferase I (CPT1A) / Abcam / EPR21843-71-2F / ab234111 / GR3351270-6 / 20
12 . anti-mouse IgG1 kappa monoclonal / Abcam / MOPC21 / ab18443 / GR3364197-6 / 200
13 . anti-mouse IgG2b kappa monoclonal / Abcam / 7E10G10 / ab170192 / GR3364314-1 / 1000
14 . Rabbit IgG monoclonal / Abcam / EPR25A / ab172730 / GR3284310-21 / 20-50
15 . Goat Anti- Mouse IgG H&L (Alexa Fluor® 647) / Abcam / n/a / ab150119 / GR3348378-3 / 2000
16 . Donkey Anti-Rabbit IgG H&L (Alexa Fluor® 647) / Abcam / n/a / ab150075 / GR3372283-8 / 2000
17 . anti-isocitrate dehydrogenase 2 (IDH2) / Abcam / EPR7577 / ab131263 / GR93077-12 / 100
18 . anti-SLC20A1 / Thermo Fisher Scientific / n/a / 12423-1-AP / 6201 / 200
19 . human-specific anti-nestin / Sigma-Aldrich / 10C2 / MAB5326 / 3822810 / 200 (tissue sections); 300 (cell culture)
20 . TOMM20 / Abcam / / ab78547 / GR3406425-1 and GR244837-3 / 400
21 . GAP43 / Abcam / EP890Y / ab75810 / GR42490-38 / 1000
22 . GFAP / Invitrogen / GA5 / 14-9892-82 / 2358413 / 200
23 . Vinculin / Invitrogen / VLN01 / MA511690 / VI3084704 / 2000
24 . F - Actin / abcam / 4E3.adl / ab130935 / GR3374194-6 / 5 mcg/mL
25 . goat-anti-mouse 647 / ThermoFisher / AB_2535804 / A-21235 / 2482945 / 200
26 . goat-anti-mouse 488 / Abcam / n/a / ab150117 / 814801 / 400
27 . goat-anti-rabbit 647 / ThermoFisher / n/a / A-21245 / 1910774 and 2299231 / 200 (tissue sections); 500 (cell culture)
28 . goat-anti-mouse HRP / Invitrogen / polyclonal / #31430 / ZC1230 / 10000
29 . goat-anti-rabbit HRP / Invitrogen / polyclonal / #31462 / 814801 / 10000
30 . rabbit-anti-phopho-histone H3 (Ser10) / Cell Signaling Technology / polyclonal / #9701 / no. 17 / 1000
31 . rabbit-anti-cleaved caspase-3 (Asp175) / Cell Signaling Technology / polyclonal / #9661 / #47 / 1000
32 . Tomm20 (for L1 and Di318 model assays) / Cell Signaling Technology / D8T4N / 42406 / no. 4 / 200
33 . anti-rabbit DyLight405 alpaca antibody / Jackson ImmunoResearch / AB_2922864 / 611-474-215 / / 300
34 . anti-actin hFAB Rhodamine / Bio-Rad / n/a / #12004163 / 64422460 / 5000
35 . anti-sox2 / R&D Systems / 245610 / MAB2018 / KGQ0319101 / 1000
36 . anti-oct4 / Cell Signaling Technology / polyclonal / #2750 / no. 5 / 1000
37 . ATP5A-AF647 / Abcam / EPR13030(B) / ab176569 / gr3399307-3 / 50
38 . GLUT1-AF647 / Abcam / EPR3915 / ab115730 / gr3406572-2 / 500

Validation

Antibody / Supplier / Cat. No / Validation
1 . chicken anti-GFP antibody / AvesLabs / GFP-1020 / Manufacturer COA detected GFP in transgenic mice (Western and microscopy)
2 . donkey anti-chicken AF488 / Jackson ImmunoResearch / 703-545-155 / Manufacturer COA reports minimal cross reactivity with non-chicken serum proteins
3 . APC-conjugated anti-CD11b antibody / Biolegend / 101212 / Manufacturer COA with appropriate staining of CD11b positive cells in mixed cel population of mouse bone marrow cells
4 . ASS1 / Abcam / ab124465 / Manufacturer COA reports appropriate band size detection by western blot across multiple cell types
5 . anti-ATP synthase F1 subunit alpha (ATP5A) / Abcam / ab110273 / Manufacturer COA reports appropriate size protein detection by western blot human/mouse cells; and appropriate localization to mitochondria on microscopy
6 . anti-glucose transporter 1 (GLUT1) / Abcam / ab115730 / Manufacturer COA reports specific binding across multiple cell lines, and no binding in knockout cells
7 . anti-glucose 6 phosphate dehydrogenase (G6PD) / Abcam / ab210702 / Manufacturer COA reports detection of appropriately sized protein on Western, and detection in appropriate cell types on histologic analysis of multiple organs
8 . anti-Acetyl-CoA carboxylase (ACC1) / Abcam / ab269273 / Manufacturer COA reports appropriate protein size detectoin by western, including for mouse and human cells, with variation of expression levels of target. Histologic analysis with appropriate cell labeling
9 . anti-peroxiredoxin 2 (PRDX2) / Abcam / ab109367 / Manufacturer COA reports appropriate band size and histologic staining across mouse and human samples. Loss of detection in target knockout cells
10 . anti-hexokinase 1 (HK1) / Abcam / ab150423 / Manufacturer COA reports appropriate band size and histologic staining across mouse and human samples. Loss of detection in target knockout cells
11 . anti-Carnitine palmitoyltransferase I (CPT1A) / Abcam / ab234111 / Manufacturer COA reports appropriate band size and histologic staining across mouse and human samples. Loss of detection in target knockout cells
12 . anti-mouse IgG1 kappa monoclonal / Abcam / ab18443 / Manufacturer COA reports lack of binding to broad array of human and mouse cell and tissue lysates
13 . anti-mouse IgG2b kappa monoclonal / Abcam / ab170192 / Manufacturer COA reports lack of binding to broad array of human and mouse cell and tissue lysates
14 . Rabbit IgG monoclonal / Abcam / ab172730 / Manufacturer COA reports lack of binding to broad array of human and mouse cell and tissue lysates
15 . Goat Anti- Mouse IgG H&L (Alexa Fluor® 647) / Abcam / ab150119 / Manufacturer COA reports reacts specifically with mouse IgG and with light chains common to other mouse immunoglobulins. No antibody was detected against non-immunoglobulin serum proteins. Reduced cross-reactivity to bovine, chicken, horse, human, pig, rabbit and rat IgG was detected. This antibody may cross react with IgG from other species.
16 . Donkey Anti-Rabbit IgG H&L (Alexa Fluor® 647) / Abcam / ab150075 / Manufacturer Coa reports specific binding to rabbit IgG with minimal cross reactivity to other immunoglobulins
17 . anti-isocitrate dehydrogenase 2 (IDH2) / Abcam / ab131263 / Manufacturer COA reports appropriate band size and histologic staining across mouse and human samples. Loss of detection in target knockout cells
18 . anti-SLC20A1 / Thermo Fisher Scientific / 12423-1-AP / Manufacturer COA reports appropriate band size and histologic staining across mouse and human samples.
19 . human-specific anti-nestin / Sigma-Aldrich / MAB5326 / Manufacturer COA and PMID 17202146 show postive signal of appropriate MW in human cells epxressing nestin. Species specificity was validated in this manuscript using rodent vs. human tissue

20 . TOMM20 / Abcam / ab78547 / Manufacturer COA demonstrates appropriate size (Western blor) and localizatoin (microscopy) of staining. We also validated TOMM20 is localized in mitochondria which was verified by colocalization with mitoDsRed.
21 . GAP43 / Abcam / ab75810 / Manufacturer COA with detection of appropriate size protein (western) and tissue distribution (microscopy) of target protein in human, mouse, and rat samples
22 . GFAP / Invitrogen / 14-9892-82 / Manufacturer COA demonstrates tissue specific (present in brain, absent in liver) of rat and mouse.
23 . Vinculin / Invitrogen / MA511690 / Manufacturer COA with advanced validation using cell lines +/- expression of vinculin
24 . F - Actin / abcam / ab130935 / Manufacturer COA reports appropriate band size (western) and cell localization on microscopy. Mouse and human cell targets
25 . goat-anti-mouse 647 / ThermoFisher / A-21235 / Manufacturer COA demonstrates appropriate staining of mouse primary antibodies and low cross-reactivity with non mouse
26 . goat-anti-mouse 488 / Abcam / ab150117 / Manufacturer COA shows minimal cross-reactivity with non-rodent IgG and appropriate staining when using a mouse primary antibody
27 . goat-anti-rabbit 647 / ThermoFisher / A-21245 / Manufacturer COA demonstrates appropriate staining vs. isotype control, and loss of staining in knockout models for target of primary antibody used in experiments
28 . goat-anti-mouse HRP / Invitrogen / #31430 / Manufacturer COA reports specific binging to mouse IgG and not other serum proteins. Minimal cross-reactivity with non-mouse IgG
29 . goat-anti-rabbit HRP / Invitrogen / #31462 / Manufacturer COA reports specific binding to rabbit immunoglobulins, no binding to non immunoglobulin serum proteins, and minimal cross-reactivity with other species
30 . rabbit-anti-phopho-histone H3 (Ser10) / Cell Signaling Technology / #9701 / Manufacturer COA with appropriate detection of phospho site upon induction of phosphorylation, not in its absence
31 . rabbit-anti-cleaved caspase-3 (Asp175) / Cell Signaling Technology / #9661 / Manufacturer COA with appropriate detection of caspase cleavage upon pharmacologic induciton of apoptosis, not in its abssence
32 . Tomm20 (for L1 and Di318 model assays) / Cell Signaling Technology / 42406 / Manufacturer COA reports minimal signal on mouse tissue. Detection of appropriate moleclar weight (western) and cellular localizaton (microscopy) in human tissue
33 . anti-rabbit DyLight405 alpaca antibody / Jackson ImmunoResearch / 611-474-215 / Manufacturer COA reports minimal cross-reactivity with non-rabbit antibodies. Positive signal when used with rabbit primary antibodies
34 . anti-actin hFAB Rhodamine / Bio-Rad / #12004163 / Manufactorer COA reports specific detection of human and rodent actin, and no cross reactivity with other secondaries
35 . anti-sox2 / R&D Systems / MAB2018 / Manufafacturer COA reports specific detection of sox2 in human, mouse, rat cells (western) and tumor specimen microscopy. Negative in non-tumor cells without sox2 expression
36 . anti-oct4 / Cell Signaling Technology / #2750 / Manufacturer COA with appropriate staining of human testicular cancer, negative in normal testes. Western with detection of appropriate sized band
37 . ATP5A-AF647 / Abcam / ab176569 / Manufacturer COA reports appropriate band size detection by western blot, with intensity varying by ATP5A expression
38 . GLUT1-AF647 / Abcam / ab115730 / Manufacturer COA reports specific binding across multiple cell lines, and no binding in knockout cells

Eukaryotic cell lines

Policy information about cell lines and Sex and Gender in Research

Cell line source(s)

1. SB28 (Dr. Hideho Okada, University of San Fransisco).
2. GL261 (Developmental Therapeutics Program, National Cancer Institute).
3. D456 patient-derived xenograft (Dr. Darrel Bigner, Duke University).
4. JX22 patient-derived xenograft (Dr. Jann Sarkaria, Mayo Clinic).
5. CSC293T (Dr. Anita Hjelmeland (co-author)).
6. Human cell lines P3, GG16, BG5 and BG7 are derived from glioblastoma patient biopsies (Dr. Hrvoje Miletic (co-author)).
7. hTERT-immortalized human astrocytes (NHA) were bought from Applied Biological Materials Inc., Vancouver, Canada, Cat# T0281

Authentication

Cell lines have been authenticated by species-specific STR marker profiling. Finger priniting of P3, GG16, BG5 and BG7 cell lines.

Mycoplasma contamination

All cell lines have tested negative for Mycoplasma spp.

Commonly misidentified lines
(See ICLAC register)

None of the cell lines in the ICLAC list of misidentified lines.

Animals and other research organisms

Policy information about studies involving animals; ARRIVE guidelines recommended for reporting animal research, and Sex and Gender in Research

Laboratory animals

C57BL/6 wildtype mice (JAX Stock #000664) were purchased as needed from Jackson Laboratory. Tg(CAG- mKate2)1Poche/J (mito::mKate2, stock #032188) mice were purchased from The Jackson Laboratory, and bred in-house. NSG mice were bred in-house. Male and female 4 to 8-week-old mice were used for experiments. 6-week old immunodeficient male and female nude-RNU rats were used in some studies.
Mice were fed standard chow (Teklad Global 18% Protein Rodent Diet, cat. No. 2913, Envigo) and filtered water ad libitum, housed in forced/filtered air isolator cages containing up to 5 mice, with 12 hour light/dark cycles; temperature is maintained at 20-26C and humidity at 30-70%.
Immunodeficient nude-RNU rats of both sexes, bred in house (Bergen, Norway) were fed a diet containing standard pellets (Sniff, V1536-000), and had access to water ad libitum, and were housed in filtered air isolator cages (Allentown type IV (Rat 1800), HEPA

	filter) with 12 hour light/dark cycles, 21?C and ca. 45% humidity.
Wild animals	This study did not involve wild animals.
Reporting on sex	This study did not analyse sex-based differences. A combination of male and female animals were utilized for all experiments, with the exception of in vivo tumor initiation studies. The reason for the latter was that we were limited in sorted cell number and our prior research indicated that tumor penetrance is overall higher in males, thus requiring fewer cells for the experiment.
Field-collected samples	This study did not involve any field collection.
Ethics oversight	All the studies were approved by the Cleveland Clinic Institutional Animal Care and Use Committee or by the Norwegian National Animal Research Authority. Study approval number for Cleveland Clinic is 2019-2179.

Note that full information on the approval of the study protocol must also be provided in the manuscript.

Flow Cytometry

Plots

Confirm that:

☒ The axis labels state the marker and fluorochrome used (e.g. CD4-FITC).

☒ The axis scales are clearly visible. Include numbers along axes only for bottom left plot of group (a 'group' is an analysis of identical markers).

☒ All plots are contour plots with outliers or pseudocolor plots.

☒ A numerical value for number of cells or percentage (with statistics) is provided.

Methodology

Sample preparation	In vivo experiments: At experiment endpoint, mice were euthanized, and resected tumors or the contralateral hemisphere were digested with 1 mg/mL collagenase IV (StemCell Technologies) and 1 mg/mL DNAse I (Roche) for 15 minutes at 37°C. Samples were strained trough a 100 µm strainer (Fisherbrand) and washed with PBS prior to staining.<br>In vitro experiments: Co-cultures of primary cells + mouse GBM cell lines were harvested by 5 min of incubation with Accutase, and washed with PBS prior to staining. Human cell line samples were seeded according to the experimental set up and attached on Matrigel in NB medium with additives. At harvest, growth medium from the samples was removed and cells were detached using pre-warmed Accutase (AC) (37 degrees C) for approx. 5 min. AC was inactivated with medium from the corresponding sample and single cell suspension (SCS) was made. Samples were then sifted through a 40 um filter and pelleted in a pre-cooled centrifuge (4 degrees C). Supernatant was removed and samples were resuspended in pre-cooled 1x PBS (4 degrees C) and kept on ice until flow cytometry.<br>Cocultures of human cell line spheroids and fetal rat brain organoids (FRBO) were harvested after 72 hours (immediately following final imaging). To secure adequate cells for practical lab work and later flow analysis, provide a mean estimate of mitochondria transfer across all samples as well as an equal number of cells in each flow sample, all cocultures from corresponding conditions were pooled in labelled 1,5 ml Eppendorf tubes. Spheroid/FRBO aggregates precipitated spontaneously. Growth medium was removed before adding pre-warmed Trypsin (37 degrees C) for approx. 5 min with regular vortexing. Trypsin was inactivated with medium from the corresponding sample and SCS was made by slight vortexing. To preserve cell numbers, samples were not filtered. Pelleting was done in a pre-cooled centrifuge (4 degrees C). Supernatant was removed and samples were resuspended in pre-cooled 1x PBS (4 degrees C) with a volume corresponding to three flow samples. Finally, the samples were distributed into three equal volumes and kept on ice until flow cytometry.<br>Brain/Tumor tissue was harvested from immunodeficient nude-RNU rats. Half the tumor (approx. 400-500 mg) was fixed for imaging analysis while the other half was dissociated into SCS using Macs Neural Dissociation Kit (P) from Miltenyi Biotec (130-092-628). All steps were done according to the manufacturers protocol. After SCS, samples were resuspended in pre-cooled 1x PBS (4 degrees C) and kept on ice until flow cytometry.<br>For staining, washed cells (in PBS) were stained with LIVE/DEAD™ Fixable Blue Dead Cell Stain Kit (Thermo Fisher Scientific) for 10 minutes on ice, and then washed with PBS+2% BSA, treated with 1:50 diluted FcR blocking reagent (Miltenyi Biotec) for 15 minutes on ice and then stained with any indicated surface antibodies in PBS+2% BSA. Samples requiring intracellular antibody staining were fixed 30 min to overnight with eBioscience FoxP3 Transcription Factor Fixation Kit, and then stained with intracellular antibody cocktail.
Instrument	BD LSR Fortessa
Software	Data collection: FACS Diva v9.0 or v8.0<br>Data analysis: FlowJo software (BD Biosciences, v10.7.2)
Cell population abundance	-Purity check of sorted samples was performed by reloading the sorted fractions to Aria II sorter and by confirming their fluorescence profile.<br>-Gene expression analysis was used as additional confirmation in sort experiment used for RNAseq.<br>-Purity of sorted astrocytes was >95%. Purity of sorted mouse tumor cell lines overall was >95% (with regard to astrocyte contamination). Purity of mKate negative mouse tumor cell lines (i.e. cells that did not uptake astrocyte mitochondria in co-culture) was >95%. Purity of mKate positive mouse tumor cell lines (i.e. cells that did uptake astrocyte mitochondria in co-culture varied from 30-45%.<br>-Purity of sorted mito::mCherry positive and negative human GBM cells from co-cultures was 80-95%<br>-Sample purity was determined by internal, technical controls for each and every experiment, thereby providing a continuous surveillance of sample fluorescence and quality. The same cells used for controls and experimental groups were

simultaneously seeded to provide single fluorescence samples. These samples were used to control for fluorescent cross-contamination and for compensation under analysis. Corresponding WT cells were used as negative controls to gauge autofluorescence and provide a non-fluorescent comparison for compensation, gating and analysis purposes. For experiments using fetal rat brain organoids (FRBO), non-transduced wild-type organoids were seeded and analyzed in parallel as negative controls. For animal experiments, WT and fluorescent cells lines corresponding to the tumor were used as controls.

Gating strategy

-FSC-A/SSC-A plots were used to gate on cells, and FSC-A/FSC-H plots were used to determine the single cells and live staining was used to exclude the dead cells. For the main readout of mKate2 or mito::mCherry fluorescence positivity, background signal was determined either by using matching tumor cells that had been co-cultured with wildtype (fluorophore negative) donor cells, or obtained from intracranial tumors implanted in wiltype mice.
-The gating strategy was developed to quantify mitochondria transfer, as defined by GFP+/DsRed- events becoming GFP+/DsRed+ events. Initially, technical controls were used for making a fluorescent-specific matrix to compensate for possible spillover which could affect results. Gates were then created by identifying live cells, single cells and GFP+/DsRed- events using the GFP-/DsRed+ control sample. These gates were applied to the group as a whole, before using the GFP+/DsRed- control sample to make a final gate defining GFP+/DsRed+ events. This gate was applied to the group as a whole, thereby creating a common gating tree for all experimental and control samples indicating live cells, single cells, GFP+/DsRed- events and GFP+/DsRed+ events. The latter was defined as total mitochondria transfer, and the frequency of parent statistic was used in subsequent analyses. In parallel, other internal parameters were analyzed to check individual experimental quality, including live cells and GFP+/DsRed- events (indicating relationship between donors (GFP-/DsRed+) and acceptors (GFP+/DsRed-) in culture). The said strategy was used on all experiments without exception.
-For GFP+/DsRed- events the boundary between "positive" and "negative" was defined at or around 103 when utilizing Comp-FITC-A (GFP) on the x-axis and SSC-A on the y-axis.
- For GFP+/DsRed+ events the boundary between "positive" and "negative" was defined by the lowest threshold which did not register any GFP+/DsRed+ events in a GFP+/DsRed- control sample. This value was persistently <102 across samples. Such strategy was employed to guarantee that acceptors receiving few mitochondria (DsRed positive in lower end of scale) would still be included for quantification of total mitochondria transfer.
- For live cells and single cells, boundaries between "positive" and "negative" cells were defined using standard gating in a SSC-A/FSC-A plot and FSC-H/FSC-A plot respectively.

☒ Tick this box to confirm that a figure exemplifying the gating strategy is provided in the Supplementary Information.
