# Supplementary material for: GAP43-dependent mitochondria transfer from astrocytes enhances glioblastoma tumorigenicity
Source: Nat Cancer. 2023 May 11;4(5):648–64. doi: 10.1038/s43018-023-00556-5 (PMC10212766; doi:10.1038/s43018-023-00556-5)
Supplement: Source Data Extended Data Fig. 6 — Unprocessed western blots. [file 43018_2023_556_MOESM18_ESM.pdf]

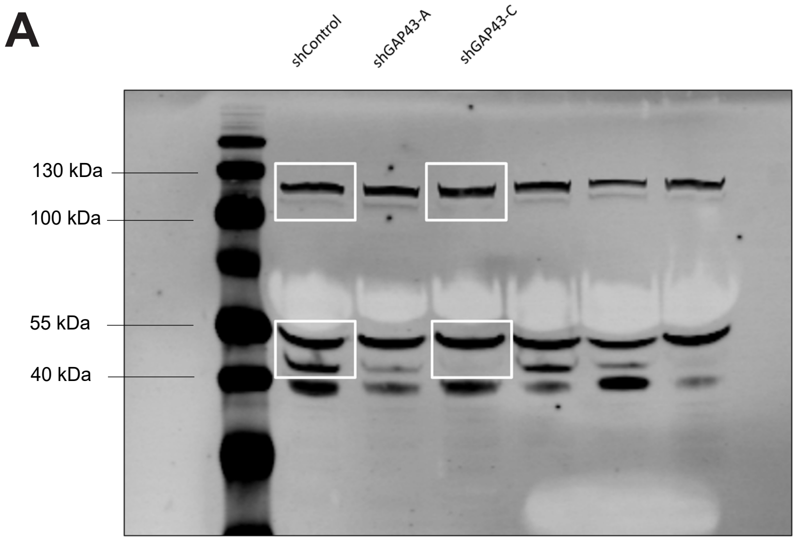

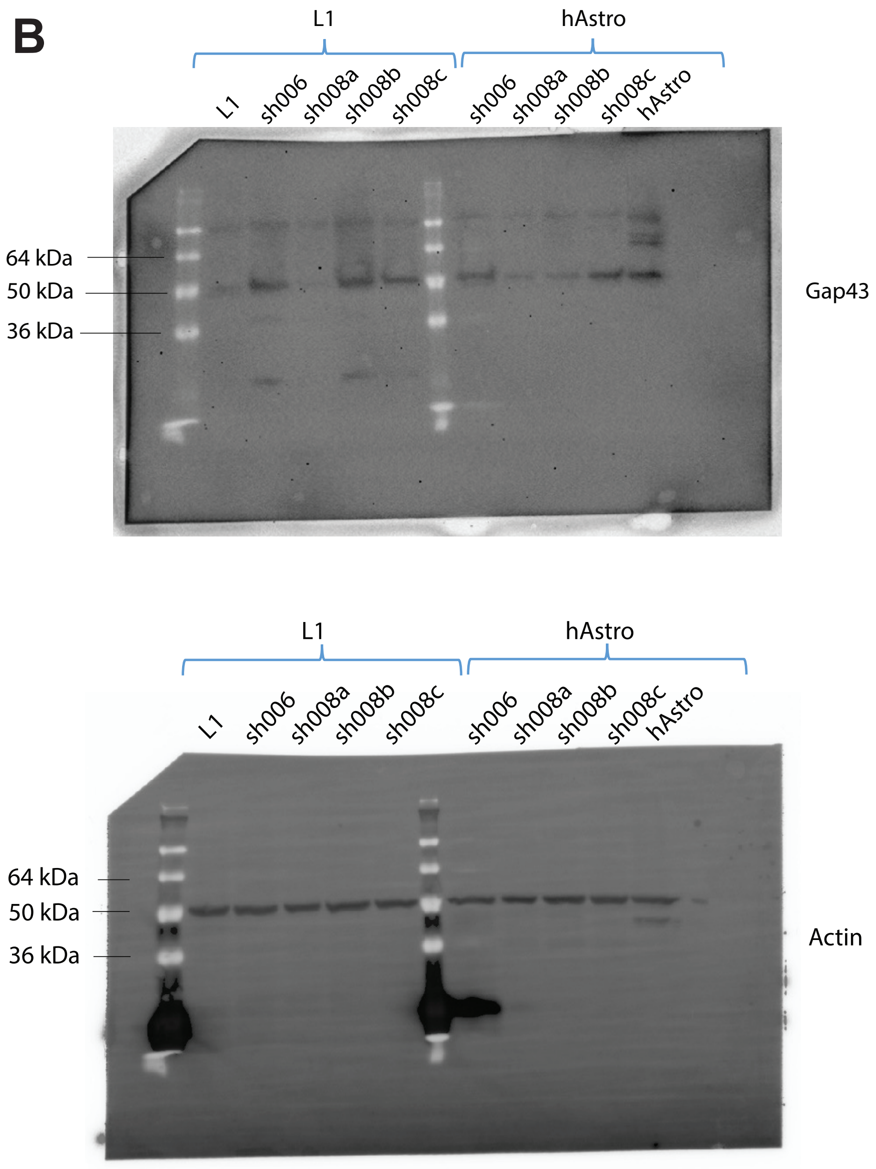

Source Data for Extended Data Fig. 6I-J

(**A**) Uncropped blot summarized in Extended Data Fig. 6I. (**B**) Uncropped blot summarized in Extended Data Fig. 6J.
