# Supplementary material for: GAP43-dependent mitochondria transfer from astrocytes enhances glioblastoma tumorigenicity
Source: Nat Cancer. 2023 May 11;4(5):648–64. doi: 10.1038/s43018-023-00556-5 (PMC10212766; doi:10.1038/s43018-023-00556-5)
Supplement: Source Data Extended Data Fig. 10 — Unprocessed western blots. [file 43018_2023_556_MOESM23_ESM.pdf]

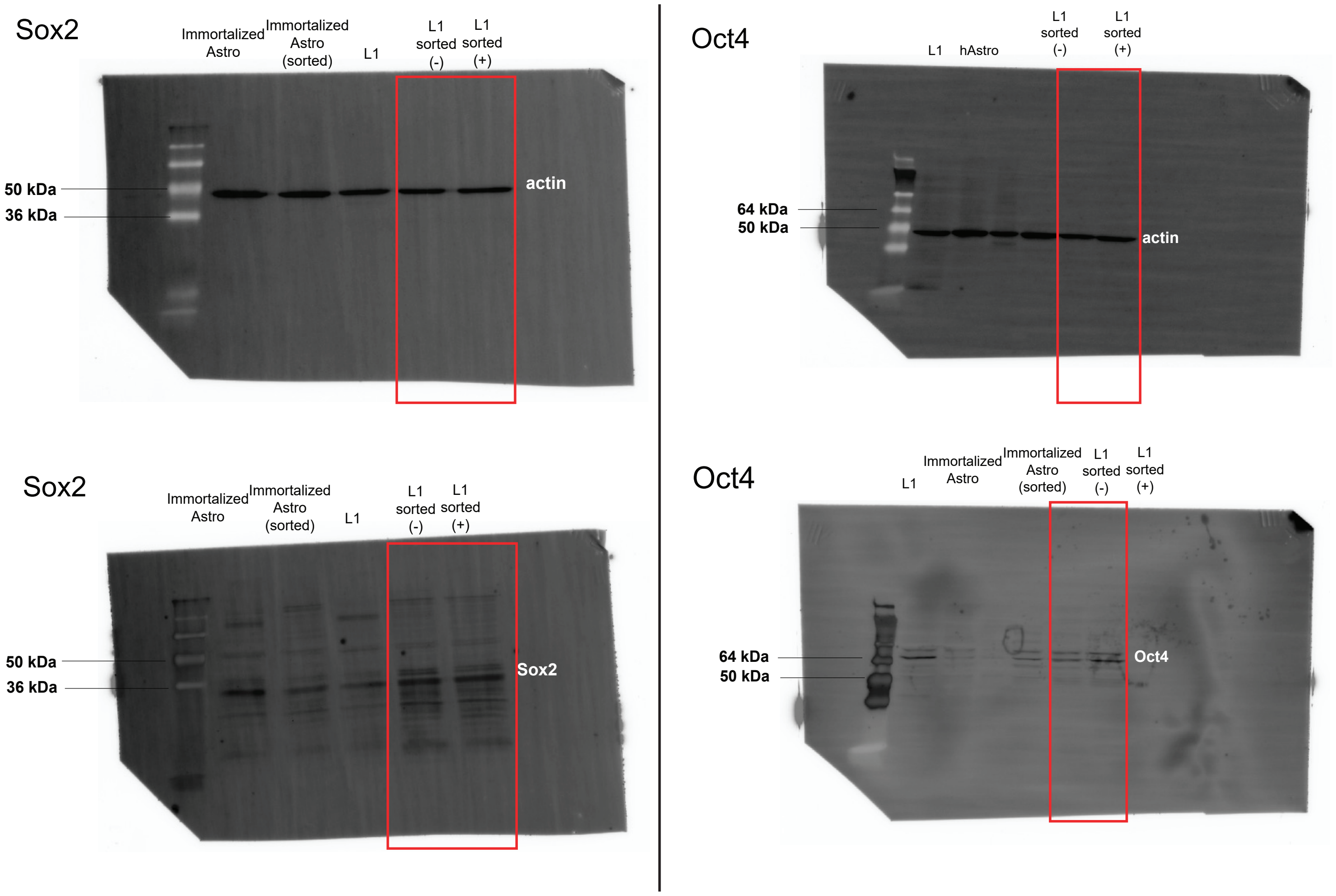

Source Data for Extended Data Fig. 10B

Uncropped Western blots for Sox2 (left) and Oct4 (right), uncluding corresponding loading control blots (actin). Red box highlights the lanes relevant to the corresponsing Extended Data figure.
